# Early adversity changes the economic conditions of mouse structural brain network organization

**DOI:** 10.1002/dev.22405

**Published:** 2023-09

**Authors:** Sofia Carozza, Joni Holmes, Petra E. Vértes, Ed Bullmore, Tanzil M. Arefin, Alexa Pugliese, Jiangyang Zhang, Arie Kaffman, Danyal Akarca, Duncan E. Astle

**Affiliations:** 1 MRC Cognition and Brain Sciences Unit, University of Cambridge, Cambridge, UK; 2 School of Psychology, University of East Anglia, Norwich, UK; 3 Department of Psychiatry, University of Cambridge, Cambridge, UK; 4 Department of Clinical Neurosciences, Wolfson Brain Imaging Centre, University of Cambridge, Cambridge, UK; 5 Bernard and Irene Schwartz Center for Biomedical Imaging, Department of Radiology, New York University School of Medicine, New York, New York, USA; 6 Department of Psychiatry, Yale University School of Medicine, New Haven, Connecticut, USA

**Keywords:** brain organization, early adversity, generative models, graph theory, structural connectome, unpredictable stress

## Abstract

Early adversity can change educational, cognitive, and mental health outcomes. However, the neural processes through which early adversity exerts these effects remain largely unknown. We used generative network modeling of the mouse connectome to test whether unpredictable postnatal stress shifts the constraints that govern the organization of the structural connectome. A model that trades off the wiring cost of long-distance connections with topological homophily (i.e., links between regions with shared neighbors) generated simulations that successfully replicate the rodent connectome. The imposition of early life adversity shifted the best-performing parameter combinations toward zero, heightening the stochastic nature of the generative process. Put simply, unpredictable postnatal stress changes the economic constraints that reproduce rodent connectome organization, introducing greater randomness into the development of the simulations. While this change may constrain the development of cognitive abilities, it could also reflect an adaptive mechanism that facilitates effective responses to future challenges.

## 1 | INTRODUCTION

The structure of the human brain undergoes complex changes over the first three decades of life (Bethlehem et al., 2022). At the macroscopic level, neural development proceeds through the formation of a network of white matter projections between populations of neurons, a process both subject to genetic control and environmental regulation (Dubois et al., 2014; Scholz et al., 2009; Stiles & Jernigan, 2010). A complete wiring map of the brain, known as a “connectome,” can be reconstructed through diffusion-weighted magnetic resonance imaging (MRI) and analyzed using graph theory (Sotiropoulos & Zalesky, 2019). Healthy neural architecture is characterized by a precise pattern of organization, or topology, that emerges over the course of childhood (Betzel, 2022; Kaiser, 2017). For instance, brain networks exhibit small-worldness, a balance between a short average path length and high clustering that permits both integrated and segregated processing of information (Bullmore & Sporns, 2009, 2012). Features of connectome organization can predict developmental differences across individuals, including variation in cognitive ability and mental health (DiMartino et al., 2014; Siugzdaite et al., 2020).

The structural organization of the brain emerges amid a tight set of constraints. The most significant of these is the spatial embedding of the network, because of which long-distance connections incur a large metabolic cost (Cherniak, 1994). The brain has adapted to limit this cost by making parsimonious use of energy and space, creating comparatively expensive features—such as connections between spatially distant regions—judiciously (Bullmore & Sporns, 2012; Chen et al., 2006). But cost minimization alone cannot account for the observed organization of biological neural networks (Costa et al., 2007; Kaiser & Hilgetag, 2004). Rather, the brain appears to negotiate an economic trade-off between the physical cost of structural connections and the topological value they add to the network (Bullmore & Sporns, 2012; Rubinov et al., 2015; Vértes et al., 2012). Recent advances in computational modeling offer a way to directly investigate the constraints that govern the development of the connectome by generating networks using different wiring rules (Bassett & Betzel, 2017; Beul et al., 2017; Horvát et al., 2016; Kaiser & Hilgetag, 2004; Vértes et al., 2012). Studies employing this approach have shown that slight manipulations in the trade-off between two key generative model terms—wiring cost and topological value—can reproduce real-world diversity in structural brain organization, and account for differences in behavioral phenotypes (Akarca et al., 2021; Betzel et al., 2016; Vértes et al., 2012). Thus, this model successfully compresses the complex topology of the biological connectomes into parameters that approximate its key features. However, the impact of developmental factors, including social environmental conditions in early life, on the wiring economy of the brain remains unknown.

The quality of the early environment is a critical determinant of neurodevelopment (McLaughlin et al., 2014). Children who experience adversity or maltreatment show subtle differences in the organization of their connectomes, including lower connectivity between modules and altered centrality of regions such as the amygdala (Ohashi et al., 2017; Teicher et al., 2016). Such neural differences may be conducive to navigating a hostile and unpredictable early environment, but may come at the expense of poorer cognition and mental health later in life (McLaughlin et al., 2019). Due to the methodological and ethical limits of human research, experimental studies in rodent models have proven invaluable for establishing the causal role of adversity in neural outcomes (Luby et al., 2020). Recent work in mice has shown that early-life stress causes local changes in brain network organization, including an increase in frontolimbic connectivity and decrease in efficiency of the amygdala, that drive a global increase in small-worldness and heightened anxiety-related behavior (Wendel et al., 2021; White et al., 2020). The increasingly thorough demonstration of adversity-related differences in brain structure highlights a crucial mechanistic gap in our understanding: how does early adversity alter the development of network-level brain organization?

In the current study, we test whether early adversity alters the wiring economy of the developing mouse connectome using a paradigm of unpredictable postnatal stress (UPS). UPS pups are raised under conditions of limited bedding to mimic impoverishment and are also exposed to unpredictable hour-long bouts of maternal separation and nest disruption to model chaotic and complex adversity (Johnson et al., 2018; White et al., 2020). We reconstructed the structural connectomes of 49 adult mice, half of which were exposed to UPS during the first 4 weeks of life (Johnson et al., 2018). Using generative network modeling, we computationally simulated realistic networks for each animal and evaluated how well they replicated the observed connectomes. We then tested for differences in the wiring economy of the brain by comparing the mathematical conditions that best replicated the connectomes of each group, and explored the developmental implications of shifts in model parameters.

## 2 | METHODS

### 2.1 | Animals

Thirty femaleBALB/cByj mice were housed in breeding cages with standard bedding, and subsequently transferred to maternity cages once visibly pregnant. On postnatal day 0 (P0), litters were culled to five to eight pups and randomly assigned to dams to mitigate the effects of genetics and litter size. Of 49 total pups, 25 (13 male and 12 female) were assigned to a control group, while 24 (12 male and 12 female) were assigned to an unpredictable early-life stress (UPS) condition. Mice in the control group were raised with standard bedding and nesting material. Mice in the UPS group received 25% of the standard amount of bedding material, received no nesting material, and were separated from their dam for 1 h on P14, P16, P17, P21, P22, and P25. Additional details about the paradigm are available elsewhere (Johnson et al., 2018). After weaning on P26, all mice were group housed with standard bedding and no nesting material. All experiments received the approval of theInstitutional Animal Care and Use Committee (IACUC) at Yale University and were conducted in accordance with the NIH Guide for the Care and the Use of Laboratory Animals.

**FIGURE 1:**
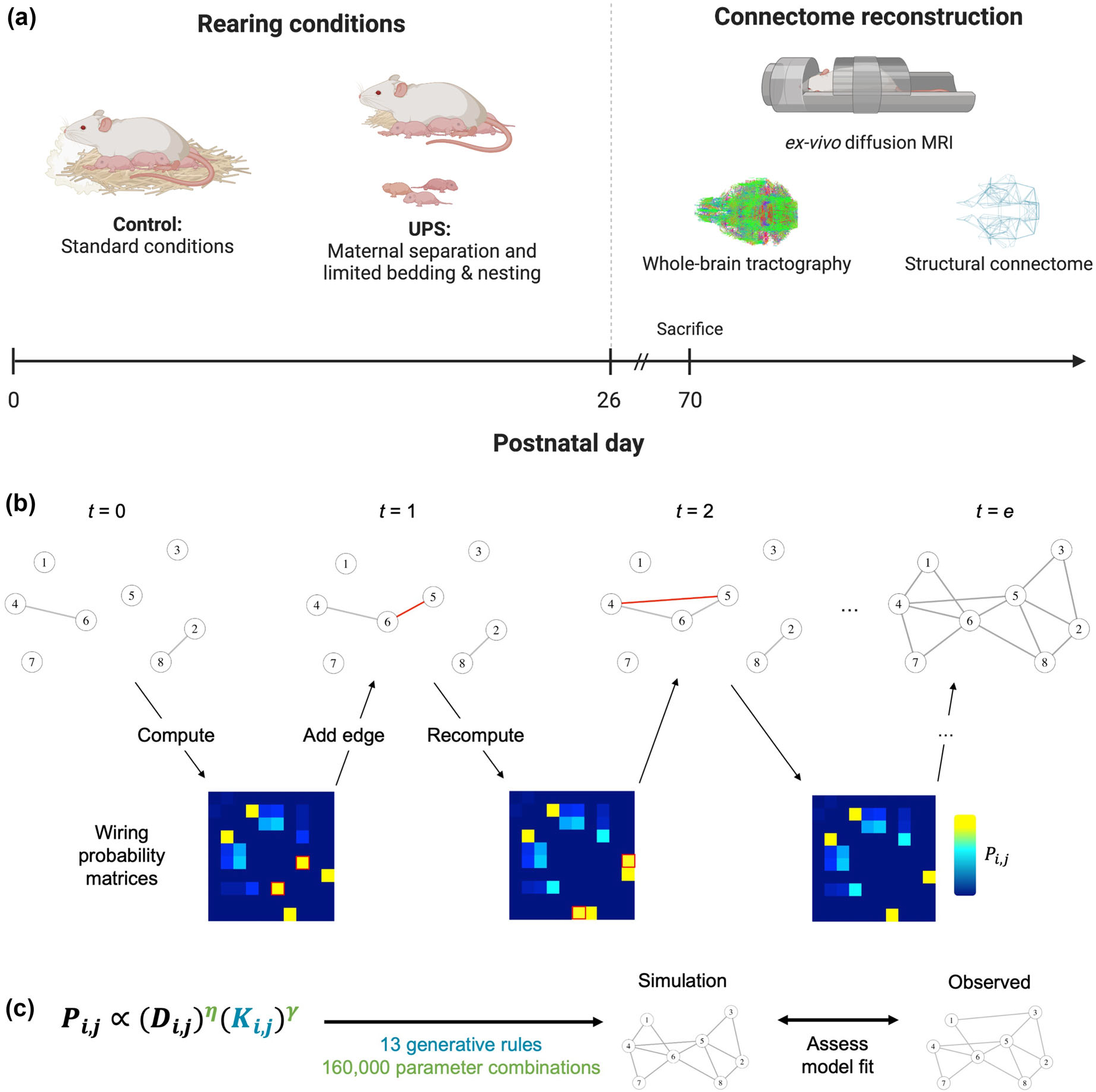
Experimental design and generative modeling procedure. (a) On postnatal day 0, 49 pups were randomly assigned to a paradigm of unpredictable postnatal stress or standard rearing conditions until postnatal day 26. After postnatal day 70, mice were sacrificed and ex vivo diffusion imaging was performed. Whole-brain probabilistic tractography was used to reconstruct the structural connectome of each animal. (b) An illustration of the generative process using a simplified connectome of 10 nodes. Starting from a sparse seed network (t=0), edges are added one at a time until the simulation reaches the number of edges found in the observed connectome (t=e). The matrix of wiring probabilities is updated at each step, allowing for dynamic shifts as the topology of the network emerges. (c) By systematically varying generative rules and parameter combinations, it is possible to identify the topological term K and the parameters η and γ that best simulate the organization of the observed connectome.

See Figure 1a for an overview of the experimental design and generative modeling procedure.

### 2.2 | Tissue and imaging acquisition

Tissue was collected from the mice in adulthood (>P70) after the conclusion of behavioral testing unrelated to this analysis. Mice were anesthetized withchloral hydrate (100 mg/kg) and, once unresponsive, transcardially perfused using cold phosphate buffer saline (PBS)/heparin (50 units/mL) solution followed by 10% formalin (polyScience). The mice were decapitated and intact skulls were immersed in 10% formalin at 4°C for 24 h, transferred to sterile 1× PBS (pH 7.4), and kept at 4°C until imaging acquisition.

Magnetic resonance images were acquired at imaging facility of New York University using a 7-Tesla scanner equipped with a cryogenic probe for enhanced signal-to-noise ratio (Ratering et al., 2008). A modified 3D gradient-and-spin-echo (3D-GRASE) sequence was used with an echo time of 33 ms, repetition time of 400 ms, 100 *μ*m isotropic resolution, two non-diffusion-weighted (b0) images, and 60 images acquired at unique gradient directions with $b=5000/{mm}^{2}$ (Wu et al., 2013). Additional acquisition details are available in a protocol paper (Arefin et al., 2021). Images were corrected for noise and Gibbs ringing artifacts using MRtrix3 (Kellner et al., 2016; Tournier et al., 2019; Veraart et al., 2016), displacement and eddy currents using FSL (Andersson & Sotiropoulos, 2016), and field bias using the N4 algorithm provided in Advanced Normalization Tools (ANTs) (Tustison et al., 2010).

### 2.3 | Connectome construction and comparisons

For each mouse, a map of brain connectivity was reconstructed using probabilistic tractography. First, unsupervised estimation of tissue-specific response functions was conducted using the Dhollander algorithm (Dhollander et al., 2016). The fiber orientation distribution was then estimated using multi-shell multi-tissue constrained spherical deconvolution (Jeurissen et al., 2014). Probabilistic streamline fiber tracking with second-order integration (iFOD2) (Tournier et al., 2010) was performed with whole-brain seeding until 10 million streamlines were reached. Fiber tracking parameters were optimized for ex vivo rodent tissue (step size, 50 *μ*m; curvature threshold, 45°; fractional anisotropy (FA) threshold, 0.1; minimum fiber length, 0.5 mm) (Chen et al., 2015; Wang et al., 2020).

A structural connectome was then built from each tractogram using a parcellation previously adapted from the Allen Mouse Brain Atlas (AMBA) and Allen Developing Mouse Brain Atlas (ADMBA) by Rubinov et al. (2015). The bilaterally symmetric parcellation consists of 41 cortical and 24 extracortical regions per hemisphere, for a total of 130 regions. Using ANTs (Avants et al., 2011), each subject image was first registered to the AMBA template space using affine and diffeomorphic transformations, then the inverse transformation was used to project the parcellation into subject space. The number of streamlines connecting each pair of regions was counted and transformed into connectivity matrices, which were symmetrized. Self-connections were removed. To eliminate spurious connections and highlight topological variation across subjects (Zalesky et al., 2016), a weight-based threshold of 6100 streamlines was applied to achieve a sparse connectome density (M=3.52%, SD=0.13%). Node-wise comparisons were conducted on four measures of local topology: nodal degree, betweenness centrality, clustering coefficient, and efficiency, each computed using the Brain Connectivity Toolbox (https://sites.google.com/site/bctnet/Home) in MATLAB. Group differences at each node were assessed using *t*-tests, and *p*-values were corrected using the False Discovery Rate approach (Benjamini & Hochberg, 1995).

The connectomes were then binarized in preparation for generative network modeling. Binary connectomes were compared between groups on five measures of global topology that have been associated with a developmental history of stress or maltreatment (Ho et al., 2018; Puetz et al., 2017): (1) number of edges; (2) total edge length, approximated using the sum of the Euclidean distances between connected regions; (3) number of long-distance edges, defined as connections that are more than 2 standard deviations above the mean connection length across the sample; (4) global efficiency, calculated as the average inverse shortest path length of the network (Bullmore & Sporns, 2009); and (5) small-worldness, defined as the ratio of clustering to shortest path length compared to its random network equivalent (Humphries et al., 2006), which was obtained by averaging across an ensemble of 500 networks that were randomized while preserving the degree distribution.

Wherever group differences were assessed, a Shapiro test was first applied to test the normality of the distributions; normal distributions were compared using analysis of variance (ANOVA), while others were compared using a Kolmogorov–Smirnov (KS) test.

### 2.4 | Generative network modeling procedure

To simulate the formation of each connectome, we formalized a trade-off between two competing factors: the wiring cost incurred by new connections and the topological value they add to the network (Betzel et al., 2016; Vértes et al., 2012). The cost term penalizes long-distance connections, thereby capturing the evolutionarily conserved drive to minimize the metabolic and material expense of axonal projections (Bassett & Betzel, 2017; Bullmore & Sporns, 2012). The value term favors connections between regions that share some topological property, such as a similar pattern of clustering or a large number of existing connections (Bassett & Betzel, 2017; Bullmore & Sporns, 2012; Kaiser, 2017).

The model begins with a preliminary seed network, which was composed of the strongest 28 connections shared across all mice so that, in line with previous work (Akarca et al., 2021; Betzel et al., 2016), it comprises about 10% of the final network density. See Figure S1 for additional details on seed network construction. At each step, the model then estimates the likelihood of a new structural connections forming within the network using the following probability equation (Betzel et al., 2016; Vértes et al., 2012):

(1) $$P_{i,j}\propto {\left(D_{i,j}\right)}^{\eta }{\left(K_{i,j}\right)}^{\gamma },$$

where $P_{i,j}$ is the probability of forming a binary connection between any two previously unconnected regions of the brain, i and j. The first term $D_{i,j}$ represents the wiring cost. As the resources required by an axonal projection increase with its length (Bullmore & Sporns, 2012), $D_{i,j}$ approximates the cost of a connection using the Euclidean distance between the brain regions it would connect. The term is scaled by a parameter η, which determines the strength of its contribution to the overall wiring probability. To penalize longer distance connections, η is negative.

The second term $K_{i,j}$ represents the topological value of a connection and can take numerous forms, each one quantifying a different topological relationship between the two nodes i and $j.\;K_{i,j}$ is scaled by a parameter γ, which is positive to favor connections with a higher topological value. Following previous work (Akarca et al., 2021; Betzel et al., 2016; Vértes et al., 2012), we tested 13 variations of K (known as “generative rules”). In addition to a purely spatial model, which did not include a topological term, we assessed two homophily models (number of common neighbors and the matching index); five clustering-based models (the average, minimum, maximum, difference in, and product of clustering coefficients); and five degree-based models (the average, minimum, maximum, difference in, and product of node degrees) (Betzel et al., 2016). All models were computed using the Brain Connectivity Toolbox (https://sites.google.com/site/bctnet/Home) in MATLAB.

At every step of the generative process, the model multiplies the cost and value terms for each pair of regions to produce a matrix of relative wiring probabilities, and probabilistically chooses a “winning” edge to add to the simulation (Figure 1b). As each added edge changes the topological similarity of certain nodes, $K_{i,j}$ and $P_{i,j}$ are continually updated at every step of the generative process. If an edge is added between nodes i and $j,\;P_{i,j}$ is set to zero. In other words, the model continually re-computes the probability of future connections.

Shifts in wiring probability can occur rapidly, especially while the connectome is sparse (Akarca et al., 2021). For example, consider the network at step t=0 in Figure 1b. Suppose it is growing according to a generative rule that favors connections between regions with shared neighbors. According to the probability function (Equation 1), nodes 4 and 5 would be unlikely to wire together at first, because they are relatively distant and share no neighbors. Instead, at step t=1, a connection forms between proximal nodes 5 and 6. However, this new connection gives nodes 4 and 5 a shared neighbor and therefore increases the topological value of forming a direct connection, which occurs at step t=2, despite the greater distance between them. While wiring cost remains the same across development, the topological value of connections, and therefore the overall wiring probability, is dynamic from one step to the next. As the network grows, longer connections become increasingly likely as the topological value added by new links outweighs the penalization of wiring cost (Akarca et al., 2022).

The generative process terminates when the synthetic network reaches the number of edges of the connectome that the model is simulating. By varying the generative rule used as the topological term $K_{i,j}$, and the η and γ parameters, it is possible to systematically manipulate the conditions that govern the development of the synthetic network and thereby to identify the rules and parameters that best simulate the connectome of an individual (Figure 1c). Thus, without modeling white matter growth, the model can shed light on what may have guided the emergence of the organization of a connectome (Akarca et al., 2021; Bassett & Betzel, 2017; Betzel et al., 2016).

### 2.5 | Evaluation of generative models

#### 2.5.1 | Model energy

To find the optimal parameters for each model, a grid search was performed in the space defined by -10≤η≤0 and 0≤γ≤10. A total of 160,000 parameter combinations were tested per subject and model, corresponding to 40,000 unique values of both η and γ.

The fit of each simulation was assessed according to the following energy equation (Betzel et al., 2016):

(2) $$E=max\left({KS}_{k},{KS}_{c},{KS}_{b},{KS}_{d}\right).$$

The equation consists of the KS statistics comparing the synthetic and empirical networks on distributions of node degree (k), clustering coefficients (c), betweenness centrality (b), and edge length (d). These four measures are critical properties of networks that are linked to stress exposure and psychiatric conditions (Ho et al., 2018; Menon, 2011) and have previously been used to assess the similarity of empirical and economically simulated connectomes (Akarca et al., 2021; Betzel et al., 2016; Zhang et al., 2021). As the energy is the maximum of the four statistics, a lower energy corresponds to better model fit, or a more similar distribution of nodal statistics between the simulated and observed networks.

### 2.6 | Model topological fingerprints

While Equation (2) compares the overall distributions of nodal statistics, it does not reveal whether a simulation replicates local patterns of relationships between these statistics. For instance, brain regions with high betweenness centrality tend to be lower in clustering, given their position between modules (Fornito et al., 2016). To assess the relative ability of generative models to capture such hallmarks of empirical connectivity, the topological fingerprint (TF) matrices of both empirical and synthetic networks were calculated.

TF matrices are a measure recently developed for this purpose (Akarca et al., 2022). First, the lowest energy simulations produced by each generative rule were selected. Six common measures of nodal topology were then calculated, including degree, betweenness centrality, clustering coefficient, edge length, local efficiency, and mean matching index. Pearson correlations between these measures were computed, averaged across the sample, and summarized in a 6 × 6 TF matrix.

A visual comparison of the synthetic and empirical TF matrices provides a heuristic for assessing the similarity of the correlational structure of their topology, and thereby evaluating the generative models’ ability to replicate the organization of empirical networks. To quantify this formally, the difference between TF matrices is given by the following equation (Akarca et al., 2022):

(3) $$\Delta {\text{TF}}_{i,j}=\sqrt{\sum_{i}\sum_{j}{\left({\text{TF}}_{{\text{empirical}}_{i,j}}-{\text{TF}}_{{\text{systhetic}}_{i,j}}\right)}^{2}}.$$

Here, ΔTF is the Euclidean norm of the difference between empirical and synthetic TF matrices. Using ΔTF, the generative rules from each category (i.e., spatial, homophily, clustering, and degree) that produced the lowest energy networks were compared. The generative rule that obtained the lowest ΔTF was used in all subsequent analyses. To obtain accurate estimates of the optimal parameters for each subject, a second grid search of an additional 40,000 parameter combinations was performed in a smaller parameter space defined by -3.75≤η≤-1.75 and 0.2≤γ≤0.6.

To extend the analysis of local topology to include the precise location of connections, a measure of the edge overlap between the synthetic and empirical networks, consisting of the sum of true positives and true negatives, was also computed.

#### 2.6.1 | Modal spatial layout

The spatial layout of the six nodal measures was also assessed (Akarca et al., 2021). Four of these measures (node degree, betweenness centrality, clustering coefficient, and edge length) are included in the energy equation, while two (local efficiency and matching index) are not. For each measure, the value at each node was averaged across the synthetic networks of all 49 subjects, resulting in a single 130 × 1 vector. The same procedure was performed on the empirical connectomes. Linear correlations between synthetic and empirical vectors were then calculated. At each node, the spatial error (or discrepancy) of each measure was calculated by subtracting its average value in the synthetic networks from its average value in the empirical connectomes (Akarca et al., 2021). Thus, a lower spatial error indicates more similarity between the local topology of a particular region in the simulations and in the observed connectomes. An absolute error was calculated as the sum of the *Z*-scores of all six generative errors.

### 2.7 | Group comparisons on generative modeling parameters

A partial least squares (PLS) discriminant analysis (Wold, 2004) was run to test whether the optimal model parameters (i.e., the values of η and γ producing the lowest energy simulations) reflected a single latent factor. The correlation between each predictor component and the primary response component was calculated, and their significance was assessed by permuting the group membership of the mice 100,000 times. For the loading of each parameter onto the PLS components, 95% confidence intervals were calculated by generating 100,000 bootstrapped samples of 49 subjects and re-computing the loadings.

The distance of each mouse from the origin of the parameter space (i.e., η=0 and γ=0) was then calculated and compared between groups using ANOVA.

### 2.8 | Exploration of model stochasticity

To explore the implications of a shift in model parameters, the composition of the wiring probability matrices $\left(P_{i,j}\right)$ was also compared between groups (Akarca et al., 2022). This was achieved by testing for differences in the distribution of probability values in the wiring matrix, taken as the average across all steps of the lowest energy simulations. To examine whether the dispersion among probability values emerges over the course of the generative process, the variance among wiring probabilities was calculated across developmental time.

To consider the effects of attenuated model parameters more systematically, additional simulations were run scaling η and γ toward zero (i.e., running models at 90%, 80%, 70%, 60%, 50%, 40%, 30%, 20%, 10%, and 0% of the optimal parameters). The distribution of values found in the wiring probability matrices $\left(P_{i,j}\right)$ of these simulations was measured and plotted. To evaluate the randomness of simulation topology, the final networks for each of these simulations were compared to 1000 randomly wired networks using the ΔTF measure described above. The same comparison was conducted using the optimal simulations for each mouse, and the biological connectomes were derived through tractography.

Previous work has suggested that early adversity accelerates the pace at which brain networks become integration and segregated over the course of childhood (Tooley et al., 2021). To assess the potential impact of model parameters on the emergence of these topological features, the trajectories of integration and segregation of the simulations across the generative process were reconstructed. The integration of the networks was operationalized as global efficiency, calculated as the average inverse shortest path length of the network (Bullmore & Sporns, 2009). The segregation of the networks was operationalized, following previous work (Tooley et al., 2020), as maximum modularity (*Q*), which quantifies the difference between the observed intracommunity connectivity and that expected by chance (Newman, 2004). The trajectories were scaled to 100 steps to present developmental time as a percentage of the total length of the generative process.

## 3 | RESULTS

### 3.1 | Empirical connectomes

Using probabilistic tractography, we reconstructed structural connectomes for each mouse. The UPS group showed higher betweenness centrality of the right visual area and left cerebellar cortex, lower betweenness centrality of the right hypothalamus and right ectorhinal cortex, and greater nodal degree and efficiency in the left auditory area (Tables S1–S4). Once binarized for generative network modeling, the connectomes showed no differences between groups on gross measures of global topology, including on number of edges (p=.89), number of long-distance connections (p=.52), maximum modularity (p=.72), global efficiency (p=.71), or small-worldness (p=.47) (see Section 2; Table S5). Groups did not differ on the distributions of key local characteristics, including node degree, clustering coefficient, betweenness centrality, edge length, mean matching index, and nodal efficiency (all p>.96) (see Section 2; Table S6).

### 3.2 | Homophily-based simulations achieve best model fit

We first sought to identify the generative rule that most successfully reproduced the structural connectomes of our sample of mice (N=49). For each animal and generative rule, we tested 160,000 parameter combinations evenly distributed throughout the space defined by -10≤η≤0 and 0≤γ≤10. We assessed how well each synthetic network fit the connectome it was simulating using the following energy equation (Betzel et al., 2016):

(4) $$E=max\left({KS}_{k},{KS}_{c},{KS}_{b},{KS}_{d}\right),$$

which compares the nodal statistics of each synthetic network to the organization of the biological connectome; if the distributions of these topological features are similar, then the energy will be low.

**FIGURE 2:**
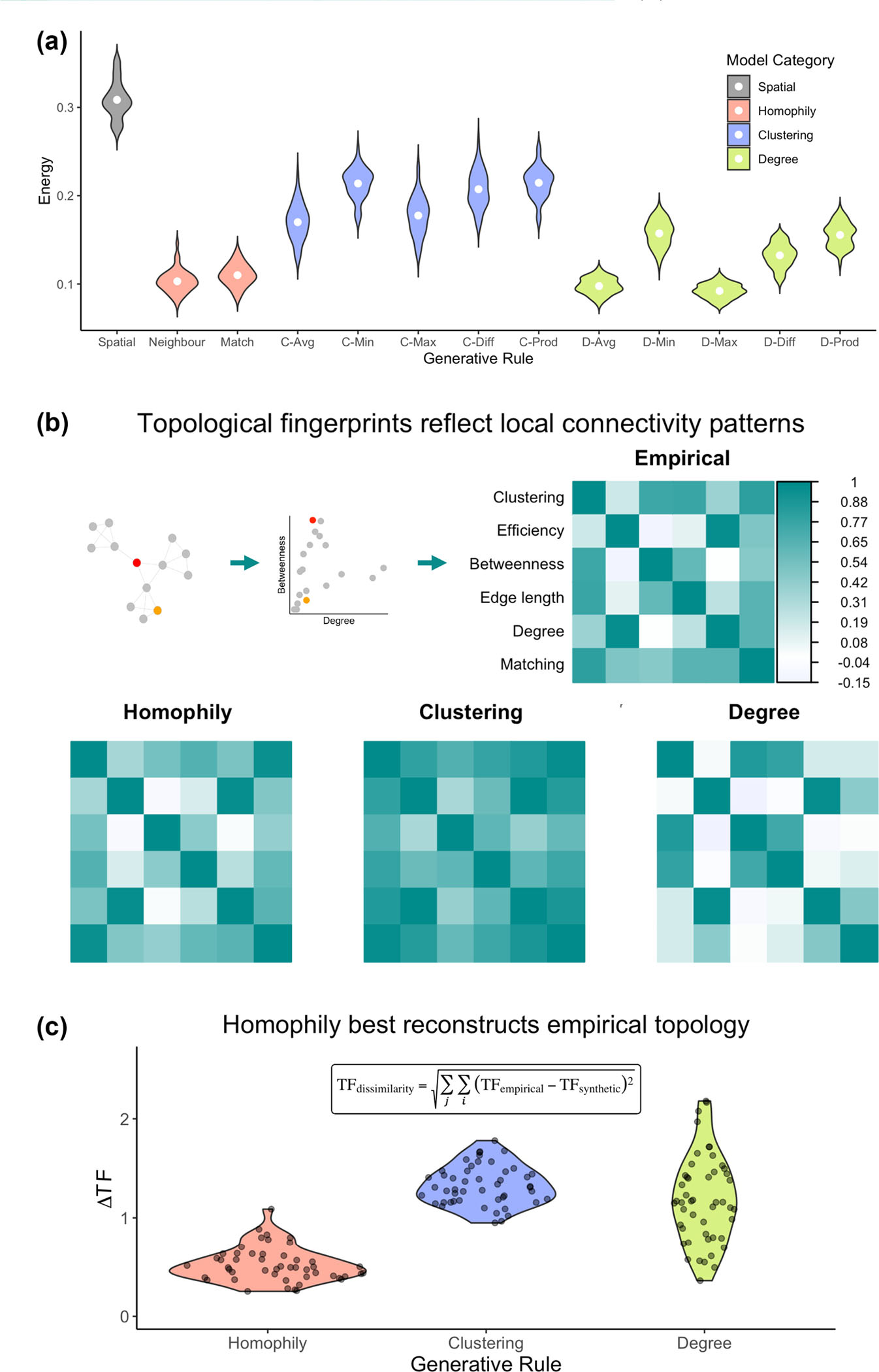
Relative performance of generative network models in replicating the organization of empirical connectomes. (a) The energy of the top-performing synthetic networks for each animal (N=49) across 13 generative rules: a purely spatial model, which considers only the distance between two regions; two homophily models, which also consider a measure of the similarity of the neighborhoods of the respective regions; five clustering-based models, which compare the clustering coefficients of the regions; and five degree-based models, which compare their node degree. White points indicate the sample mean. (b) The topological fingerprint (TF) is a correlation matrix of local network statistics, including node degree, clustering coefficient, betweenness centrality, total edge length, local efficiency, and mean matching index. TFs are shown for the empirical networks and the best-performing rules across the three categories of generative models. Across all four matrices, the value of the correlation can be inferred from the color bar (spans −0.15 [pale lilac] through 0 [white] to 1 [teal]). Correlations shown are the sample average (N=49). (c) Across the sample (N=49), homophily achieves lowest ΔTF, a measure of the discrepancy between the correlational structure of the local topology of the simulations and the empirical connectomes, computed using the equation shown.

To assess the performance of the models, we compared the lowest energy simulation produced by each rule. All generative rules outperformed a purely spatial model that considered only wiring cost (Figure 2a; Table S7). An ANOVA and post hoc Tukey test confirmed that models specifying homophily as the topological $K_{i,j}$ term achieved lower energy than those utilizing clustering ($diff\;=\;-0.090,\;p=1.97\times 10^{-12}$) or degree ($diff\;=\;-0.020,\;p=1.16\times 10^{-9}$). To account for the stochastic nature of network development, we repeated this comparison while taking the average of the energy of the top 10 best-performing networks for each subject. Though the difference in group means shrank, homophily once again outperformed the clustering-based ($diff\;=\;-0.082,\;p=1.97\times 10^{-12}$) and degree-based ($diff\;=\;-0.012,\;p=6.95\times 10^{-4}$) categories of models (Figure S2). Thus, generative models that trade off the wiring cost of a connection with a measure of neighborhood similarity produce synthetic networks whose global topological distributions closely resemble those of the observed connectomes. As multiple models achieved low energy, the success of the top-performing models from each category was examined further.

### 3.3 | Homophily best recapitulates the local properties of observed networks

The energy equation effectively assesses how closely the statistical distributions of nodal characteristics of the synthetic networks resemble those of the empirical connectomes. However, brain networks also exhibit local patterns of relationships between nodal characteristics. To assess whether the models successfully captured this, we calculated the TF matrices (Akarca et al., 2022) of the empirical and simulated networks, a measure that summarizes the unique patterns of local organization found across a network (see Section 2).

**FIGURE 3:**
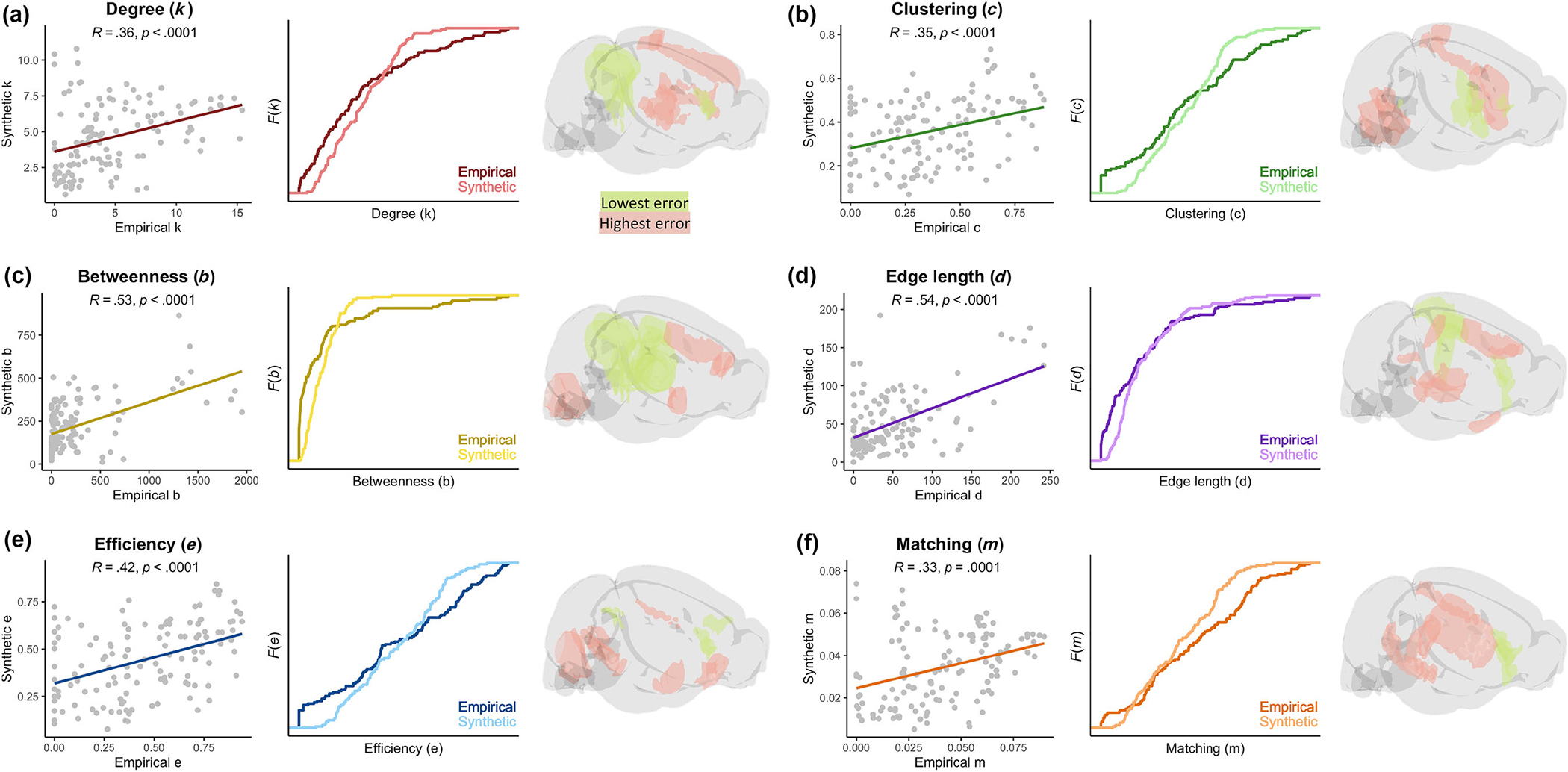
Simulated networks replicate spatial layout of empirical connectomes. Each point in the scatterplots represents the nodal measure for one of the 130 regions of the parcellation, taken as the average value across animals (N=49). For each of the six measures, a significant positive correlation exists between the nodes of synthetic and empirical networks. A cumulative density function of the measure is also displayed, as well as a visualization of the mouse brain in which the five regions with the lowest and highest error (i.e., discrepancy between synthetic and empirical networks) are highlighted in green and red, respectively. Four of the statistics ([a] node degree, [b] clustering coefficient, [c] betweenness centrality, and [d] total edge length) are terms of the energy equation used to assess the fit of the synthetic networks, while the remaining statistics ([e] local efficiency and [f] mean matching index) are not.

TFs for the empirical connectomes and the top-performing generative models from each category can be found in Figure 2b (all other rules are shown in Figure S3a). An ANOVA with post hoc Tukey test indicated that the homophily model achieved lower ΔTF than the best clustering-based ($diff\;=\;-0.804,\;p<1.0\times 10^{-20}$) and degree-based models ($diff\;=\;-0.662,\;p<1.0\times 10^{-20}$), confirming the visual impression that its TF most resembled that of the empirical connectomes (Figure 2c; comparable results are shown for all other rules in Figure 3b). In other words, a model that balances the cost of an additional connection against the number of shared neighbors produces networks with local patterns of organization that closely resemble those of the rodent connectome, even though local topology was not explicitly optimized by the energy function.

Note that similarity in local network organization does not necessarily entail exact correspondence in edge location. An ANOVA with post hoc Tukey test found that the lowest energy homophily model achieved lower edge overlap than the lowest energy clustering model ($diff\;=\;-67.35,\;p=1.05\times 10^{-10}$) but did not detect a difference between it and the lowest energy degree model (diff=3.18,p=0.99) (Figure S4). Thus, while the homophily-based approach bests approximate topology, this may not extend to the precise localization of edges.

### 3.4 | Homophily replicates spatial layout of empirical networks

Given that the wiring of biological neural networks is shaped by their embedding in anatomical space (Bassett & Stiso, 2018), realistic synthetic connectomes should ideally exhibit a spatial layout of topology akin to that of connectomes derived from tractography. To test this similarity, we first calculated the six characteristics of each node of the parcellation, averaged across the sample, then correlated the values between simulated and empirical connectomes (Akarca et al., 2021; Betzel et al., 2016). As shown in Figure 3, all four measures included in the energy equation exhibited significant correlations: degree ($r\;=\;.360,\;p=2.68\times 10^{-5}$), clustering coefficient ($r\;=\;.346,\;p=5.40\times 10^{-5}$), betweenness centrality ($r\;=\;.530,\;p=9.33\times 10^{-11}$), and edge length ($r\;=\;.543,\;p=2.52\times 10^{-11}$). Correlations were also observed between synthetic and empirical nodes on local efficiency ($r\;=\;.420,\;p=7.01\times 10^{-7}$) and mean matching index ($r\;=\;.334,\;p=1.02\times 10^{-4}$). When repeating the assessment of spatial similarity between empirical and simulated connectomes at the individual level, the correlations on these measures were more moderate and variable across the sample (degree: M=0.1948,SD=0.1353; M=0.0946,SD=0.0808; M=0.1857,SD=0.1233;M=0.2923,SD=0.01325; M=0.1411,SD=0.0932; M=0.1686,SD=0.1787). Thus, for some subjects and for the sample on average, the simulations replicated the spatial layout of key nodal features.

We also assessed discrepancies between the simulated and observed connectomes in the layout of these local characteristics. At each node, we computed a measure of spatial error by subtracting the average value of each characteristic in the synthetic networks from its average value in the empirical connectomes (Akarca et al., 2021). Thus, a lower spatial error indicates more similarity between the local topology of a particular region in the simulations and in the observed connectomes. While overall spatial error was distributed throughout the brain (Table S8), a significant correlation was observed between spatial error and node degree in the seed network ($r\;=\;.436,\;p=2.221\times 10^{-7}$) (Table S9). This indicates that generative models may benefit from instructions as to where to begin adding connections if they are to best replicate the spatial patterning of network characteristics.

### 3.5 | Early adversity attenuates wiring constraints in optimal simulations

**FIGURE 4:**
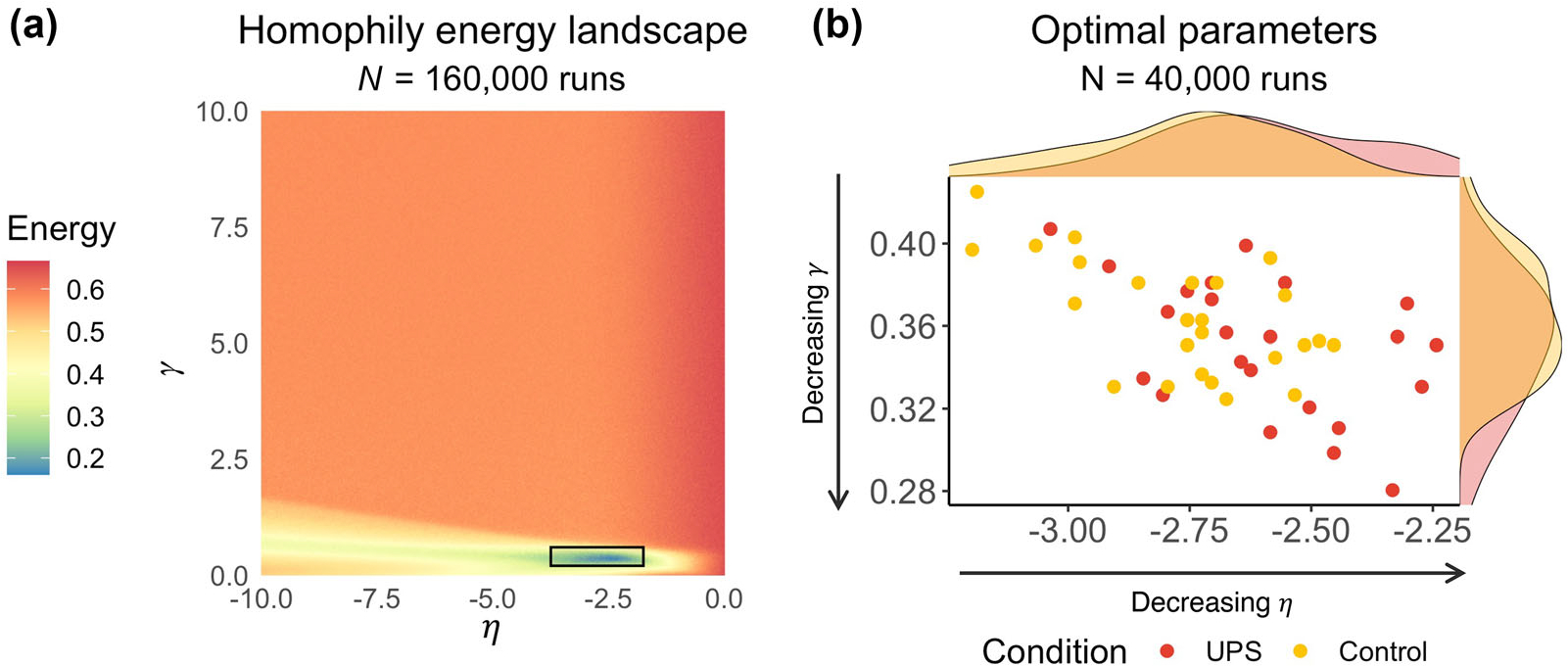
Adversity attenuates optimal generative modeling parameters. (a) In the first run of the homophily model, 160,000 unique combinations of cost parameter η and value parameter γ were tested. The energy surface shown is the sample average (N=49). (b) Optimal values of η and γ produce the lowest energy synthetic networks. Values were obtained by testing an additional 40,000 parameter combinations in a narrow low-energy window of the initial grid search, highlighted with a black rectangle in panel (a). Each data point in the scatterplot represents a single animal. Density plots above and to the right highlight differences between unpredictable postnatal stress (UPS) and control conditions. Optimal parameters tend to fall closer to the origin—at the bottom right of the plot—for animals in the UPS condition (ANOVA $F_{1,\;47}=5.700,\;p=.021$).

Across all generative models, the homophily model implementing the neighbor rule exhibited the smallest coefficient of variation in the γ parameter and second smallest in the η parameter (Table S7). Thus, while this rule was best able to account for variations in topology across animals, it did so through minute adjustments in the weighting of its cost and value terms, likely indicative of the highly regulated nature of connectomic organization (Figure 4a). To obtain maximally precise parameters for each animal, we therefore performed a second search of 40,000 parameter combinations in a narrow space centered at the apparent minimum of the energy landscape: -3.75≤η≤-1.75 and 0.2≤γ≤0.6.

The parameters producing the lowest energy networks for each animal are shown in Figure 4b. The cost and value parameters were correlated ($r=-.574,\;p=1.65\times 10^{-5}$), placing the simulations on an axis from the origin of the parameter space (η=0,γ=0). This indicates that simulations with a more severe penalty on long-distance connections usually had stronger preference for connections between regions with shared neighbors.

Along this axis, animals in the UPS condition tended to fall closer to the origin; we confirmed this observation by comparing the length of a vector from the origin to each point between groups (UPS M=2.63,SD=0.213, Control M=2.79,SD=0.210; ANOVA $F_{1,\;47}=5.700,\;p\;=\;.021$). The simulations for animals in the UPS condition were therefore subject to weaker constraints on the formation of connections. One possible confound here is that the models may simply perform better for one group than the other, but this was not the case: no difference in model energy was observed (UPS M=0.101,SD=0.015, Control M=0.105,SD=0.010; ANOVA $F_{1,47}=0.719,\;p\;=\;.401$). To test the sensitivity of this finding to the choice of the lowest energy simulation, we took the average of the eta and gamma across the 10 lowest energy simulations (Figure S5). While the UPS group was found closer to the origin of the parameter space (UPS M=2.68,SD=0.117, Control M=2.71,SD=0.106), the difference in group means again shrank, and an ANOVA did not detect a significant difference (ANOVA $F_{1,\;47}=1.185,\;p\;=\;.282$).

What is the nature of this group difference in parameters? One possibility is that either or both parameters drive the change in a relatively independent manner. Alternatively, it could reflect a single underlying shift in wiring constraints that incorporates both parameters. We distinguished these alternatives using a PLS discriminant analysis (see Section 2). This formally tests for the presence of underlying factors that explain the group difference in parameter combinations. There was a significant correlation between the group affiliation and the first latent variable ($r=.36,\;p_{\text{permuted}}=.011$) but not the second latent variable $\left(p_{\text{permuted}}=.898\right)$. Both parameters of the generative model (η coefficient =−1.5549, 95% confidence interval [CI] [−1.8929, −1.2969]; γ coefficient = 0.1229, 95% CI [0.0684, 0.1858]) loaded significantly onto this component. There was no between-group difference in scores on the component (KS $D_{1,\;47}=0.308,\;p\;=\;.159$). Thus, it seems that the observed group difference in location in the parameter space reflects a change that incorporates both wiring parameters, rather than reflecting one or two independent effects.

### 3.6 | Shift in wiring economy induces greater stochasticity

Simulations closer to the origin of the parameter space have greater stochasticity or randomness in the generative process (Akarca et al., 2022). To understand why this is the case, imagine that the edges in the wiring probability matrix are competing with one another. When the cost penalty and topological preferences are strong, fewer edges have high probabilities of wiring and the preferred winner is clear. But when constraints are weaker, more edges qualify as good contenders, giving the probabilistic nature of the process a greater role in the gradual organization of the network.

**FIGURE 5:**
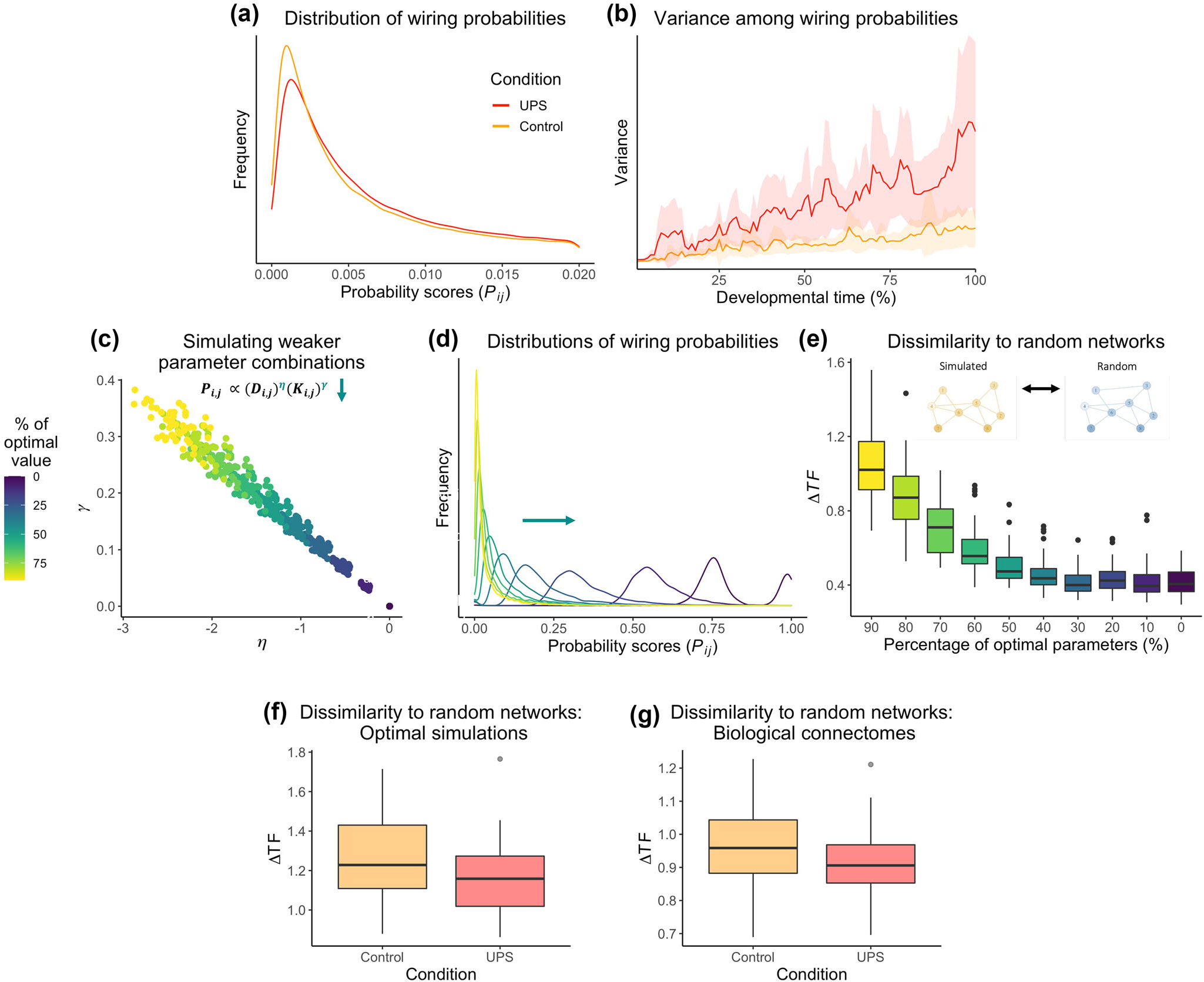
Weaker wiring constraints heighten stochasticity of network development. (a) Distributions of wiring probabilities $\left(P_{i,j}\right)$ within the probability matrix, taken as the group averages across all steps of optimal simulations. The unpredictable postnatal stress (UPS) condition shows a flatter distribution with greater dispersion, corresponding to more connections with higher wiring probabilities. (b) Variance among values in the probability matrix $\left(P_{i,j}\right)$ corresponds to the dispersion of likelihoods of potential future connections. Wiring probability variance rises as simulations develop, especially in the UPS condition, indicating that model stochasticity was more pronounced later in the process. (c) To assess the effect of systematically manipulating wiring constraints, simulations were run at 10% increments from the optimal values for each animal to zero. This resulted in the 490 parameter combinations plotted in this space. (d) Distributions of wiring probabilities $\left(P_{i,j}\right)$ within the probability matrix, taken as the average across all steps, at each parameter interval. Wiring probabilities for simulations with weaker parameters approach a normal distribution. (e) Topological dissimilarity (ΔTF; see Section 2) was averaged across 1000 randomly wired networks. The organization of simulated networks gradually resembles random topology as parameters approach zero. The same trend is observed when comparing the UPS condition to the control condition, both for (f) optimal generative models and (g) biological connectomes derived through tractography.

Simulations for the UPS condition showed a flatter distribution with a greater dispersion of values in the probability matrix compared to the control condition (Figure 5a; KS $D_{1,\;47}=0.055,\;p=2.20\times 10^{-16}$), corresponding to more potential connections with higher probabilities of wiring and therefore heightened stochasticity. Variance among wiring probabilities rose over the course of the development of each simulation, particularly in the UPS condition, indicating that this increase in stochasticity was more pronounced later in the generative process (Figures 5b and S6). At the end of the generative process, the simulations for mice in the UPS condition exhibited a distribution of node degree that was closer to normal (kurtosis: KS $D_{1,47}\;=\;0.475,\;p=.005$), indicating that the shift in wiring probabilities subtly randomized network topology.

To explore the relationship between model stochasticity and parameters more systematically, we produced additional simulations scaling η and γ toward the origin of the parameter space. Specifically, we ran models at 90%, 80%, 70%, 60%, 50%, 40%, 30%, 20%, 10%, and 0% of the optimal wiring parameters (Figure 5c). We then measure and plotted the distribution of values found in the wiring probability matrices $\left(P_{i,j}\right)$ of these simulations. As the parameters neared η=0 and γ=0, the distribution of values within the wiring probability matrices $\left(P_{i,j}\right)$ exhibited greater dispersion (Figure 5d). This corresponds to a greater number of connections with high probability of wiring over the course of the generative process.

Simulations with smaller wiring parameters had a more random topology (Figure 5e), as measured by the average ΔTF to 1000 randomly wired networks. We found the same trend toward random network topology in the UPS group, both in the optimal simulations (Figure 5f; UPS M=1.18,SD=0.214, Control M=1.27,SD=0.214, ANOVA $F_{1,\;47}=2.158,\;p\;=\;.148$) and the biological connectomes (Figure 5g; UPS M=0.915,SD=0.125, Control M=0.973,SD=0.126, ANOVA $F_{1,\;47}=2.647,\;p\;=\;.110$). Though subtle, this is in line with the principle that weaker wiring constraints heighten stochasticity in the formation of structural connections, thereby leading to more random brain network topology.

As recent work suggests that early adversity can accelerate developmental trajectories of network connectivity (Callaghan & Tottenham, 2016; Tooley et al., 2021), we reconstructed the emergence of network integration and segregation across the generative process for both the optimal simulations and the additional models run with parameters scaled toward the origin of the parameter space. Results can be found in Figure S7. Attenuated model parameters appear to accelerate the increase in global efficiency and the drop in maximum modularity that occur over the course of simulation development.

## 4 | DISCUSSION

We explored the effects of early adversity on the development of the structural connectome. We deployed generative network modeling in a mouse model of UPS to test whether adversity alters the economic trade-off that best simulates structural brain organization. The parameters that reproduced the rodent connectomes were closer to zero for the mice exposed to UPS, resulting in greater variability in wiring probabilities and therefore stochasticity in the generative process. Thus, exposure to a chaotic and unpredictable environment may attenuate or weaken constraints governing the emergence of complex brain network topology. These results point to a crucial intermediate level of explanation for the developmental impact of early adversity.

Replicating prior work in generative network modeling, models with a topological term outperformed that based purely on distance (Akarca et al., 2021; Betzel et al., 2016; Vértes et al., 2012; Zhang et al., 2021), and models implementing the principle of homophily produced the most realistic structural connectomes in terms of topology (Akarca et al., 2021; Betzel et al., 2016; Vértes et al., 2012; Zhang et al., 2021). These findings accord well with previous research on the development of the mouse brain; wiring cost alone is not sufficient to recapitulate the complex topology of its macroscopic structural networks (Rubinov et al., 2015). In our study, the neighbor rule—which favors connections between regions with a greater number of shared neighbors—produced networks that possessed not only similar statistical distributions of nodal characteristics, but also their local organization and spatial layout. Importantly, this organization was not hard-coded into the algorithm but emerged from the trade-off between cost and value over the course of the generative process. Our study is the first to implement the two-parameter generative model in rodents and replicate the comparative success of homophily in this species—though studies in humans have found that the matching index performs better than the neighbor rule, perhaps indicating a species difference in network organization (Akarca et al., 2021; Betzel et al., 2016; Vértes et al., 2012). One potential explanation for the topological success of homophily-based rules may be that they capture the impact of macroscopic dynamics of Hebbian learning: as regions with similar neighborhoods are likely to experience comparable patterns of stimulation, mechanisms of activity-dependent plasticity would favor their consolidation into a structural network (Ganguly & Poo, 2013; Vértes et al., 2014).

The parameters that produced the best-fitting synthetic networks differed between mice according to their exposure to early adversity. Specifically, simulations for mice in the UPS condition were subject to a more moderate penalty on long-distance connections and a weaker preference for connections between regions with shared neighbors. The negative correlation between model parameters, in line with previously findings (Akarca et al., 2021; Zhang et al., 2021) (but see also Betzel et al., 2016), indicates the wiring economy of the brain differs across individuals through co-variance of the two constraints. However, it is still possible that a single parameter accounts for the observed group difference. Using a PLS discriminant analysis, we confirmed that a single latent factor that incorporates both the cost penalty and value term best explains the relationship between model parameters and group affiliation. As evolutionary pressures have favored heightened phenotypic plasticity in harsh, unpredictable environments, even when this is energetically costly (Ellis et al., 2017; Frankenhuis & Amir, 2021), the brain may respond to early unpredictable stress by attenuating overall constraints on the formation of new structural connections. This finding is particularly striking because, using global measures of topology, we did not detect an effect of early adversity on structural connectivity, in contrast to previous work (Johnson et al., 2021; Kim et al., 2019; Ohashi et al., 2017; Puetz et al., 2017). While this could be due to inadequate power or the choice of experimental paradigm, it may also reveal that a generative modeling approach can approximate complex and subtle outcomes of adversity by reducing many measures of neural organization to a single latent factor.

As lower magnitude wiring parameters correspond to heightened model stochasticity, early adversity appears to favor more random structural organization of the brain. Given that UPS mice show impaired fear learning (White et al., 2020) and weaker wiring constraints are associated with poorer cognitive abilities in children (Akarca et al., 2021), our results might therefore offer a mechanistic account for the previous finding that growing up in an unpredictable environment can hamper cognitive development (Davis et al., 2017). However, greater stochasticity in network development may also reflect an advantageous process of adaptation, as individuals exposed to early adversity tend to show skills and abilities that are conducive to successfully navigating stressful contexts (Ellis et al., 2017). Across scales, the probabilistic development of neural tissue harnesses stochastic and noisy processes to build circuits that are robust to perturbation (Hiesinger & Hassan, 2018). In an adverse or unpredictable environment, heightened stochasticity in the development of the structural connectome could be adaptive if it enables the nervous system to respond more effectively to future challenges in hostile environments (Frankenhuis & Amir, 2021). This proposal is consistent with a recent finding that the connectomes of children with cognitive difficulties are more robust to random attacks on networks hubs (Siugzdaite et al., 2020).

It is important to note that our generative models are not intended to model white matter growth and not provide conclusive evidence of longitudinal differences in neural development (Bassett & Betzel, 2017). Furthermore, the group difference in parameters was small and sensitive to averaging across numerous simulations, indicating a need to replicate the effect in an independent sample. Replication should also assess the robustness of the results to different thresholding approaches. These efforts may be aided by a recently developed parameter estimation method that efficiently negotiates a trade-off between the computational burden of generative models and the accuracy and reliability of optimal parameters (Liu et al., 2023). More broadly, an exploration of the impact of the intrinsic stochasticity of the model—by conducting within- and between-parameter comparisons of its output—would be useful. Methodological developments could also increase the biological correspondence of the simulations in a few key ways. First, as the binarization of the connectomes is a gross simplification, a generative modeling strategy that produces weighted networks would be a welcome next step. This would also aid future efforts to improve the models by elaborating the model fitting process, so that it considers the location and strength of precise edges in addition to network topology (which has hitherto been the focus). Second, models may benefit from varying rules and parameters across space and time. Additionally, models could incorporate other facts known to shape connectomic organization, such as the functional identity or morphology of regions (Pathak et al., 2020; Song et al., 2014). As UPS can have sex-specific effects on brain structure (White et al., 2020), future work should test for sex differences in the wiring economy of the brain. Finally, comparing the effects of UPS to the impact of early adversity on the wiring economy of the human brain would confirm the generalizability of these results across species.

In conclusion, we found that UPS changes the economic conditions that reproduce the macroscopic structural organization of the brain. Our results offer a promising and mathematically specified path toward understanding how early life adversity contributes to diversity in structural brain network organization.

## Supplementary Material

Supplemental information

## Data Availability

Generative network modeling and analyses of synthetic networks were conducted in MATLAB, and visualizations were produced using RStudio for R. All code is available on the Open Science Framework (OSF) at osf.io/evgw5. Imaging data are available upon request to the authors. Structural connectivity matrices for each animal can be found on the OSF at osf.io/evgw5. For the purpose of open access, the author has applied a Creative Commons Attribution (CC BY) license to any Author-Accepted Manuscript version arising from this submission.
