# Supplementary material for: Early adversity changes the economic conditions of mouse structural brain network organization
Source: Dev Psychobiol. 2023 Jun 20;65(6):e22405. doi: 10.1002/dev.22405 (PMC10505050; doi:10.1002/dev.22405)
Supplement: Supplementary file 1 — Supp Information [file DEV-65-0-s001.docx]

*
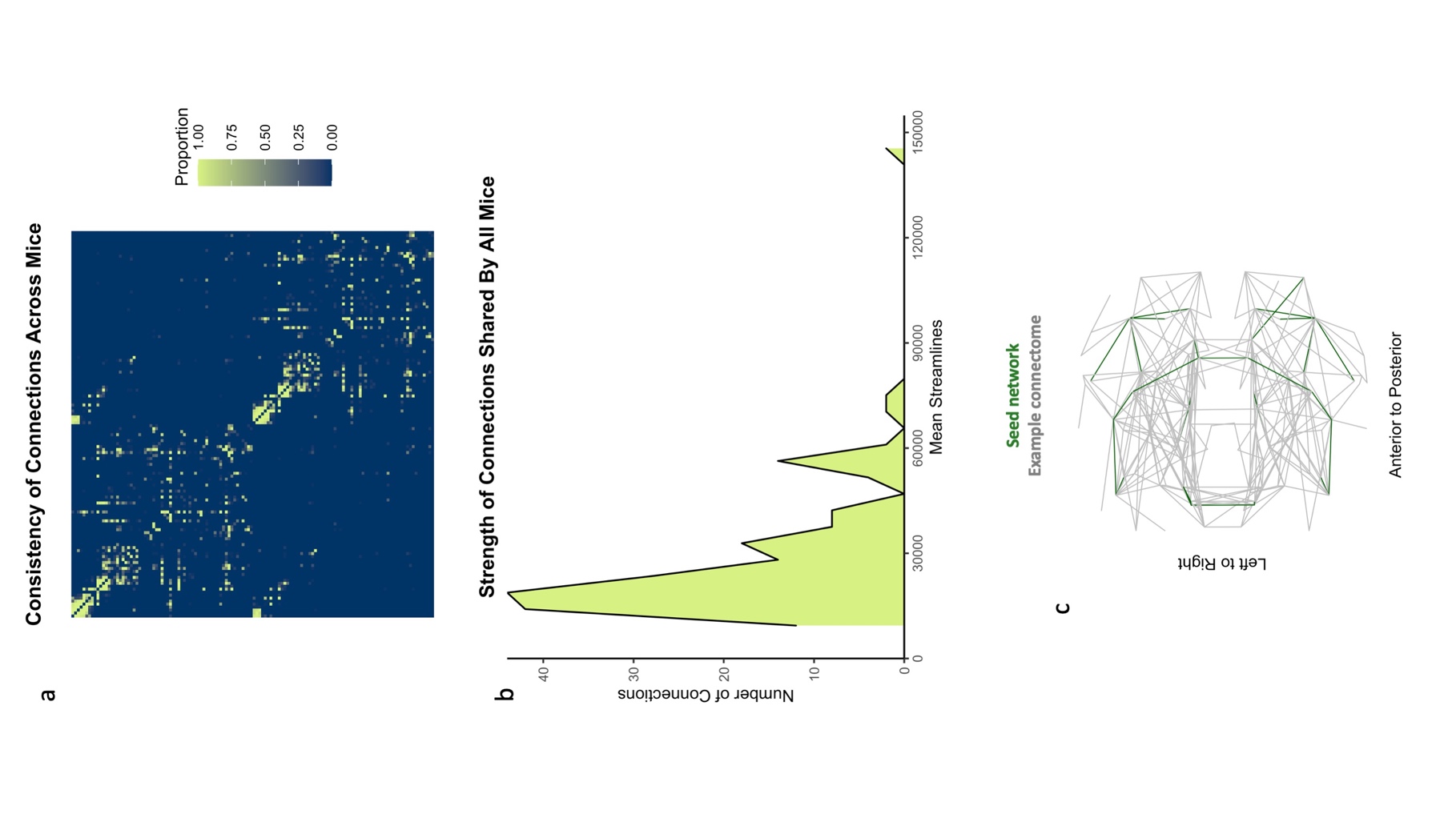
***Figure S1.** **Seed network used for generative modelling. (a)** An adjacency matrix of the 130 regions of the parcellation. The colour indicates the proportion of the sample whose connectomes contain each connection, ranging from 0 (blue) to 1 (green). **(b)** The sample mean of the weight of connections shared by all empirical connectomes. **(c)** A schematic representation of the seed network (green) superimposed over a representative empirical connectome (grey).

**Table S1. Node-wise comparisons of the nodal degree of empirical connectomes.** Nodal features were compared between UPS and control conditions using ANOVAs, and p-values were corrected using the False Discovery Rate method. For details about each region, see [1].

| **Node name** | **UPS**  ***M (SD)*** | | **Control**  ***M (SD)*** | | ***F* statistic** | **adjusted *p*** |
| --- | --- | --- | --- | --- | --- | --- |
| L-AMd | 1.29 | (0.86) | 1.20 | (0.91) | 0.13 | 0.819 |
| L-SSp-m2/3 | 3.75 | (1.11) | 3.64 | (0.99) | 0.13 | 0.819 |
| L-AUDd6a | 1.54 | (0.93) | 2.08 | (1.00) | 3.81 | 0.481 |
| L-SSp-n4 | 3.79 | (1.28) | 3.92 | (1.29) | 0.12 | 0.819 |
| L-ml | 0.08 | (0.28) | 0.08 | (0.28) | 0.00 | 0.873 |
| L-AOBmi | 2.42 | (0.83) | 2.20 | (0.71) | 0.97 | 0.708 |
| L-TEa1 | 1.88 | (1.54) | 1.72 | (1.28) | 0.15 | 0.819 |
| L-COApm1-3 | 7.17 | (1.58) | 6.48 | (2.02) | 1.74 | 0.621 |
| L-PVHpml | 11.17 | (1.71) | 11.36 | (1.60) | 0.17 | 0.819 |
| L-CUL4gr | 12.88 | (2.51) | 12.76 | (1.90) | 0.03 | 0.873 |
| L-oct | 2.42 | (1.25) | 3.00 | (1.53) | 2.13 | 0.621 |
| L-VM | 4.38 | (1.28) | 4.72 | (1.43) | 0.79 | 0.762 |
| L-FLmo | 0.54 | (0.59) | 0.32 | (0.48) | 2.11 | 0.621 |
| L-VISal6a | 0.92 | (0.72) | 0.76 | (0.72) | 0.58 | 0.784 |
| L-SCdw | 0.54 | (0.59) | 0.56 | (0.58) | 0.01 | 0.873 |
| L-RSPagl6a | 0.08 | (0.28) | 0.08 | (0.28) | 0.00 | 0.873 |
| L-TR2 | 4.75 | (1.11) | 4.56 | (1.04) | 0.38 | 0.811 |
| L-FLgr | 1.46 | (0.51) | 1.32 | (0.48) | 0.97 | 0.708 |
| L-MPT | 3.17 | (1.34) | 2.96 | (1.17) | 0.33 | 0.811 |
| L-DR | 0.88 | (0.85) | 0.92 | (0.76) | 0.04 | 0.873 |
| L-AHNp | 2.25 | (1.03) | 1.88 | (0.97) | 1.67 | 0.621 |
| L-lotd | 0.21 | (0.59) | 0.04 | (0.20) | 1.83 | 0.621 |
| L-EPd | 1.58 | (1.02) | 1.36 | (1.11) | 0.54 | 0.801 |
| L-MPNm | 0.08 | (0.28) | 0.16 | (0.37) | 0.65 | 0.773 |
| L-INC | 4.33 | (1.55) | 4.80 | (1.47) | 1.17 | 0.706 |
| L-ECT6a | 2.00 | (1.32) | 2.44 | (0.96) | 1.79 | 0.621 |
| L-CENT3gr | 7.96 | (1.88) | 8.24 | (1.51) | 0.34 | 0.811 |
| L-cbt | 0.38 | (0.58) | 0.36 | (0.70) | 0.01 | 0.873 |
| L-ORB1 | 0.00 | (0.00) | 0.00 | (0.00) | NaN | NaN |
| L-AHNa | 13.54 | (1.56) | 13.72 | (1.67) | 0.15 | 0.819 |
| L-HPF | 2.88 | (0.95) | 2.56 | (0.82) | 1.55 | 0.621 |
| L-VAL | 1.17 | (0.64) | 1.16 | (0.47) | 0.00 | 0.873 |
| L-COAa | 11.29 | (2.03) | 11.20 | (2.02) | 0.03 | 0.873 |
| L-PARN | 1.25 | (0.53) | 1.16 | (0.47) | 0.39 | 0.811 |
| L-NOD | 3.08 | (1.10) | 3.80 | (1.61) | 3.29 | 0.485 |
| L-RSPd | 6.75 | (1.73) | 7.08 | (2.02) | 0.38 | 0.811 |
| L-arb | 3.04 | (1.33) | 2.64 | (1.44) | 1.02 | 0.708 |
| L-IV | 1.96 | (0.75) | 1.84 | (0.62) | 0.36 | 0.811 |
| L-AUDv | 1.88 | (1.08) | 0.92 | (0.64) | 14.39 | 0.047 |
| L-TTd1-4 | 0.00 | (0.00) | 0.00 | (0.00) | NaN | NaN |
| L-KF | 1.00 | (1.02) | 1.32 | (1.18) | 1.03 | 0.708 |
| L-DORpm | 3.75 | (1.22) | 3.52 | (1.36) | 0.39 | 0.811 |
| L-lab | 4.17 | (1.31) | 4.32 | (1.41) | 0.16 | 0.819 |
| L-ptf | 2.96 | (1.27) | 2.48 | (1.36) | 1.62 | 0.621 |
| L-ttp | 4.21 | (1.06) | 4.32 | (1.70) | 0.08 | 0.849 |
| L-grv of CBX | 4.04 | (1.30) | 3.80 | (1.35) | 0.41 | 0.811 |
| L-VISpl6a | 8.67 | (1.74) | 8.32 | (2.34) | 0.34 | 0.811 |
| L-SSp-un | 5.33 | (1.43) | 5.32 | (1.82) | 0.00 | 0.873 |
| L-PBme | 11.25 | (2.49) | 13.00 | (2.20) | 6.82 | 0.282 |
| L-MH | 2.04 | (1.57) | 2.04 | (1.31) | 0.00 | 0.881 |
| L-IXn | 3.08 | (1.72) | 3.72 | (2.07) | 1.37 | 0.659 |
| L-VISpm4 | 7.00 | (2.02) | 6.72 | (1.51) | 0.30 | 0.814 |
| L-cbp | 10.67 | (1.97) | 10.08 | (2.16) | 0.98 | 0.708 |
| L-GU1 | 13.13 | (1.39) | 12.48 | (1.53) | 2.37 | 0.604 |
| L-MSC | 4.67 | (0.87) | 4.36 | (0.64) | 2.00 | 0.621 |
| L-ORBm2/3 | 15.17 | (1.81) | 15.24 | (1.20) | 0.03 | 0.873 |
| L-SSp-bfd1 | 0.63 | (0.49) | 0.44 | (0.51) | 1.67 | 0.621 |
| L-DMHv | 3.71 | (0.86) | 4.00 | (1.15) | 1.00 | 0.708 |
| L-DECgr | 6.63 | (1.61) | 7.04 | (1.59) | 0.82 | 0.761 |
| L-CU | 5.79 | (0.98) | 5.92 | (1.15) | 0.18 | 0.819 |
| L-CA1sp | 10.63 | (1.41) | 11.00 | (1.19) | 1.02 | 0.708 |
| L-MO6a | 5.63 | (0.88) | 5.92 | (0.76) | 1.59 | 0.621 |
| L-VISam5 | 8.79 | (1.18) | 8.80 | (1.15) | 0.00 | 0.873 |
| L-CBXmo | 7.83 | (0.76) | 7.88 | (0.33) | 0.08 | 0.849 |
| L-PB | 7.33 | (1.81) | 8.44 | (1.47) | 5.53 | 0.319 |
| R-AMd | 1.67 | (1.05) | 1.52 | (1.08) | 0.23 | 0.819 |
| R-SSp-m2/3 | 4.00 | (1.25) | 3.52 | (1.26) | 1.79 | 0.621 |
| R-AUDd6a | 1.83 | (1.17) | 2.00 | (0.91) | 0.31 | 0.814 |
| R-SSp-n4 | 3.79 | (0.98) | 3.88 | (1.20) | 0.08 | 0.849 |
| R-ml | 0.17 | (0.38) | 0.16 | (0.37) | 0.00 | 0.873 |
| R-AOBmi | 2.58 | (1.18) | 2.44 | (0.82) | 0.25 | 0.819 |
| R-TEa1 | 2.25 | (1.59) | 1.72 | (1.28) | 1.66 | 0.621 |
| R-COApm1-3 | 7.88 | (1.60) | 7.24 | (2.26) | 1.28 | 0.682 |
| R-PVHpml | 11.75 | (2.42) | 11.28 | (1.79) | 0.60 | 0.781 |
| R-CUL4gr | 14.17 | (2.71) | 13.00 | (2.25) | 2.69 | 0.570 |
| R-oct | 2.33 | (1.34) | 2.60 | (1.41) | 0.46 | 0.811 |
| R-VM | 5.71 | (1.60) | 4.52 | (1.23) | 8.54 | 0.282 |
| R-FLmo | 0.83 | (0.70) | 0.64 | (0.76) | 0.86 | 0.755 |
| R-VISal6a | 1.08 | (0.78) | 1.36 | (0.99) | 1.17 | 0.706 |
| R-SCdw | 0.54 | (0.72) | 0.64 | (0.57) | 0.28 | 0.815 |
| R-RSPagl6a | 0.04 | (0.20) | 0.04 | (0.20) | 0.00 | 0.873 |
| R-TR2 | 4.92 | (1.25) | 4.48 | (1.12) | 1.66 | 0.621 |
| R-FLgr | 1.58 | (0.58) | 1.32 | (0.56) | 2.61 | 0.570 |
| R-MPT | 3.38 | (1.17) | 2.72 | (0.84) | 5.07 | 0.359 |
| R-DR | 0.96 | (0.75) | 0.92 | (0.86) | 0.03 | 0.873 |
| R-AHNp | 2.25 | (1.07) | 2.12 | (0.97) | 0.20 | 0.819 |
| R-lotd | 0.21 | (0.41) | 0.12 | (0.33) | 0.68 | 0.773 |
| R-EPd | 1.54 | (0.88) | 1.60 | (1.04) | 0.04 | 0.873 |
| R-MPNm | 0.04 | (0.20) | 0.04 | (0.20) | 0.00 | 0.873 |
| R-INC | 4.50 | (1.69) | 5.64 | (1.47) | 6.35 | 0.282 |
| R-ECT6a | 2.46 | (1.28) | 3.04 | (1.06) | 3.00 | 0.527 |
| R-CENT3gr | 8.83 | (1.43) | 9.60 | (1.53) | 3.27 | 0.485 |
| R-cbt | 0.42 | (0.65) | 0.44 | (0.82) | 0.01 | 0.873 |
| R-ORB1 | 0.00 | (0.00) | 0.00 | (0.00) | NaN | NaN |
| R-AHNa | 14.46 | (1.44) | 15.04 | (1.95) | 1.40 | 0.658 |
| R-HPF | 3.08 | (0.97) | 2.56 | (0.77) | 4.38 | 0.446 |
| R-VAL | 1.50 | (0.72) | 1.20 | (0.41) | 3.24 | 0.485 |
| R-COAa | 10.88 | (2.56) | 11.40 | (1.80) | 0.69 | 0.773 |
| R-PARN | 1.50 | (0.66) | 1.20 | (0.41) | 3.70 | 0.481 |
| R-NOD | 3.04 | (1.04) | 3.72 | (1.49) | 3.40 | 0.485 |
| R-RSPd | 6.46 | (1.69) | 6.48 | (1.66) | 0.00 | 0.873 |
| R-arb | 3.04 | (1.23) | 3.00 | (1.47) | 0.01 | 0.873 |
| R-IV | 2.13 | (0.95) | 1.52 | (0.77) | 6.04 | 0.282 |
| R-AUDv | 1.88 | (1.19) | 1.80 | (1.00) | 0.06 | 0.869 |
| R-TTd1-4 | 0.00 | (0.00) | 0.00 | (0.00) | NaN | NaN |
| R-KF | 0.92 | (0.83) | 1.36 | (1.11) | 2.48 | 0.590 |
| R-DORpm | 4.63 | (1.21) | 4.32 | (1.22) | 0.78 | 0.762 |
| R-lab | 5.46 | (1.79) | 5.24 | (1.39) | 0.23 | 0.819 |
| R-ptf | 3.58 | (1.61) | 3.80 | (1.71) | 0.21 | 0.819 |
| R-ttp | 4.50 | (1.29) | 4.32 | (1.22) | 0.25 | 0.819 |
| R-grv of CBX | 3.58 | (1.10) | 3.76 | (1.23) | 0.28 | 0.815 |
| R-VISpl6a | 8.96 | (1.57) | 8.48 | (2.42) | 0.67 | 0.773 |
| R-SSp-un | 5.33 | (1.69) | 5.12 | (1.74) | 0.19 | 0.819 |
| R-PBme | 11.08 | (2.22) | 12.84 | (2.58) | 6.50 | 0.282 |
| R-MH | 2.25 | (1.45) | 2.12 | (1.13) | 0.12 | 0.819 |
| R-IXn | 2.79 | (1.44) | 3.48 | (1.42) | 2.83 | 0.551 |
| R-VISpm4 | 7.58 | (1.79) | 6.96 | (1.74) | 1.52 | 0.621 |
| R-cbp | 10.42 | (1.82) | 10.00 | (1.66) | 0.70 | 0.773 |
| R-GU1 | 13.83 | (1.90) | 13.48 | (2.16) | 0.37 | 0.811 |
| R-MSC | 4.79 | (1.02) | 4.84 | (0.90) | 0.03 | 0.873 |
| R-ORBm2/3 | 15.50 | (1.50) | 15.28 | (1.14) | 0.34 | 0.811 |
| R-SSp-bfd1 | 0.83 | (0.38) | 0.80 | (0.41) | 0.09 | 0.849 |
| R-DMHv | 4.63 | (1.13) | 4.40 | (0.87) | 0.61 | 0.781 |
| R-DECgr | 7.42 | (1.67) | 7.92 | (1.15) | 1.52 | 0.621 |
| R-CU | 6.21 | (1.06) | 6.76 | (0.83) | 4.12 | 0.446 |
| R-CA1sp | 11.00 | (1.10) | 10.96 | (0.84) | 0.02 | 0.873 |
| R-MO6a | 5.88 | (0.85) | 5.96 | (0.61) | 0.16 | 0.819 |
| R-VISam5 | 8.88 | (0.80) | 8.68 | (1.11) | 0.50 | 0.811 |
| R-CBXmo | 7.63 | (0.71) | 8.00 | (0.58) | 4.12 | 0.446 |
| R-PB | 7.63 | (1.71) | 8.76 | (1.16) | 7.40 | 0.282 |

[1] Wiring cost and topological participation of the mouse brain connectome. Mikail Rubinov, Rolf J. F. Ypma, Charles Watson, Edward T. Bullmore.

Proceedings of the National Academy of Sciences Aug 2015, 112 (32) 10032-10037; DOI: 10.1073/pnas.1420315112.

**Table S2. Node-wise comparisons of the nodal betweenness centrality of empirical connectomes.** Nodal features were compared between UPS and control conditions using ANOVAs, and p-values were corrected using the False Discovery Rate method. For details about each region, see [1].

| **Node name** | **UPS**  ***M (SD)*** | | **Control**  ***M (SD)*** | | ***F* statistic** | **adjusted *p*** |
| --- | --- | --- | --- | --- | --- | --- |
| L-AMd | 16.75 | (39.83) | 12.24 | (36.62) | 0.17 | 0.183 |
| L-SSp-m2/3 | 337.33 | (584.71) | 290.88 | (402.07) | 0.11 | 0.193 |
| L-AUDd6a | 230.50 | (469.71) | 323.44 | (361.35) | 0.61 | 0.157 |
| L-SSp-n4 | 164.92 | (192.55) | 108.16 | (125.47) | 1.51 | 0.138 |
| L-ml | 0.00 | (0.00) | 0.00 | (0.00) | NaN | NaN |
| L-AOBmi | 193.50 | (312.43) | 104.72 | (207.43) | 1.38 | 0.138 |
| L-TEa1 | 214.08 | (364.82) | 75.20 | (172.91) | 2.94 | 0.128 |
| L-COApm1-3 | 1000.00 | (753.13) | 872.88 | (683.27) | 0.38 | 0.161 |
| L-PVHpml | 845.42 | (447.09) | 897.12 | (561.45) | 0.13 | 0.191 |
| L-CUL4gr | 1773.42 | (1008.17) | 1252.00 | (689.63) | 4.50 | 0.117 |
| L-oct | 61.42 | (232.20) | 34.64 | (85.19) | 0.29 | 0.166 |
| L-VM | 271.67 | (234.18) | 324.88 | (314.49) | 0.45 | 0.157 |
| L-FLmo | 2.33 | (11.43) | 0.00 | (0.00) | 1.04 | 0.138 |
| L-VISal6a | 7.42 | (22.85) | 1.84 | (9.20) | 1.27 | 0.138 |
| L-SCdw | 13.42 | (65.73) | 8.80 | (44.00) | 0.08 | 0.193 |
| L-RSPagl6a | 0.00 | (0.00) | 0.00 | (0.00) | NaN | NaN |
| L-TR2 | 519.17 | (377.96) | 498.96 | (312.15) | 0.04 | 0.193 |
| L-FLgr | 42.00 | (156.39) | 8.16 | (40.80) | 1.09 | 0.138 |
| L-MPT | 154.67 | (189.20) | 204.16 | (242.16) | 0.63 | 0.157 |
| L-DR | 24.50 | (77.49) | 24.16 | (66.58) | 0.00 | 0.211 |
| L-AHNp | 300.92 | (464.82) | 141.92 | (188.81) | 2.50 | 0.137 |
| L-lotd | 2.50 | (12.25) | 0.00 | (0.00) | 1.04 | 0.138 |
| L-EPd | 52.67 | (147.71) | 16.16 | (59.74) | 1.31 | 0.138 |
| L-MPNm | 0.00 | (0.00) | 0.00 | (0.00) | NaN | NaN |
| L-INC | 198.25 | (229.32) | 254.00 | (325.24) | 0.48 | 0.157 |
| L-ECT6a | 300.00 | (432.36) | 362.16 | (370.13) | 0.29 | 0.166 |
| L-CENT3gr | 608.42 | (513.64) | 751.60 | (506.87) | 0.96 | 0.144 |
| L-cbt | 9.17 | (44.91) | 2.56 | (12.80) | 0.50 | 0.157 |
| L-ORB1 | 0.00 | (0.00) | 0.00 | (0.00) | NaN | NaN |
| L-AHNa | 1341.08 | (368.27) | 1474.56 | (473.43) | 1.21 | 0.138 |
| L-HPF | 61.58 | (98.05) | 38.32 | (90.19) | 0.75 | 0.157 |
| L-VAL | 35.75 | (76.95) | 22.64 | (58.19) | 0.45 | 0.157 |
| L-COAa | 532.17 | (502.67) | 363.68 | (336.51) | 1.92 | 0.138 |
| L-PARN | 110.25 | (379.06) | 12.32 | (41.89) | 1.65 | 0.138 |
| L-NOD | 31.25 | (64.68) | 83.76 | (135.98) | 2.94 | 0.128 |
| L-RSPd | 680.50 | (501.86) | 796.72 | (619.86) | 0.52 | 0.157 |
| L-arb | 151.42 | (186.49) | 137.68 | (155.57) | 0.08 | 0.193 |
| L-IV | 117.92 | (211.46) | 68.08 | (112.24) | 1.07 | 0.138 |
| L-AUDv | 84.00 | (172.79) | 23.36 | (80.85) | 2.51 | 0.137 |
| L-TTd1-4 | 0.00 | (0.00) | 0.00 | (0.00) | NaN | NaN |
| L-KF | 10.00 | (46.47) | 8.88 | (28.54) | 0.01 | 0.200 |
| L-DORpm | 97.17 | (78.06) | 159.44 | (158.31) | 3.01 | 0.128 |
| L-lab | 309.42 | (241.36) | 333.12 | (363.08) | 0.07 | 0.193 |
| L-ptf | 215.75 | (320.18) | 191.92 | (243.47) | 0.09 | 0.193 |
| L-ttp | 468.00 | (433.23) | 640.88 | (541.90) | 1.51 | 0.138 |
| L-grv of CBX | 104.50 | (201.35) | 48.40 | (111.51) | 1.47 | 0.138 |
| L-VISpl6a | 409.17 | (396.52) | 336.56 | (228.71) | 0.62 | 0.157 |
| L-SSp-un | 717.25 | (764.42) | 593.52 | (533.50) | 0.43 | 0.157 |
| L-PBme | 709.17 | (579.87) | 1028.16 | (626.31) | 3.42 | 0.128 |
| L-MH | 21.58 | (73.59) | 17.20 | (29.07) | 0.08 | 0.193 |
| L-IXn | 51.83 | (123.52) | 94.80 | (161.06) | 1.09 | 0.138 |
| L-VISpm4 | 345.58 | (306.76) | 423.68 | (410.55) | 0.57 | 0.157 |
| L-cbp | 769.17 | (602.87) | 963.60 | (1128.23) | 0.56 | 0.157 |
| L-GU1 | 1333.42 | (679.95) | 1107.92 | (846.01) | 1.05 | 0.138 |
| L-MSC | 160.50 | (366.72) | 71.12 | (153.75) | 1.26 | 0.138 |
| L-ORBm2/3 | 1429.00 | (730.00) | 1528.16 | (867.95) | 0.19 | 0.182 |
| L-SSp-bfd1 | 0.00 | (0.00) | 0.00 | (0.00) | NaN | NaN |
| L-DMHv | 203.50 | (255.73) | 106.16 | (115.23) | 2.99 | 0.128 |
| L-DECgr | 391.42 | (508.81) | 377.04 | (354.08) | 0.01 | 0.199 |
| L-CU | 347.17 | (233.69) | 270.48 | (246.30) | 1.25 | 0.138 |
| L-CA1sp | 319.50 | (208.30) | 253.92 | (185.15) | 1.36 | 0.138 |
| L-MO6a | 122.25 | (259.71) | 109.68 | (208.30) | 0.04 | 0.193 |
| L-VISam5 | 258.33 | (277.45) | 301.12 | (358.04) | 0.22 | 0.177 |
| L-CBXmo | 108.58 | (100.45) | 44.56 | (58.09) | 7.54 | 0.057 |
| L-PB | 161.50 | (170.31) | 209.28 | (248.23) | 0.61 | 0.157 |
| R-AMd | 77.92 | (164.04) | 49.04 | (108.98) | 0.53 | 0.157 |
| R-SSp-m2/3 | 378.58 | (582.92) | 119.52 | (210.39) | 4.35 | 0.117 |
| R-AUDd6a | 110.67 | (207.15) | 230.32 | (419.92) | 1.58 | 0.138 |
| R-SSp-n4 | 135.00 | (195.31) | 132.32 | (218.53) | 0.00 | 0.207 |
| R-ml | 0.00 | (0.00) | 0.00 | (0.00) | NaN | NaN |
| R-AOBmi | 255.67 | (350.85) | 138.64 | (212.85) | 2.01 | 0.138 |
| R-TEa1 | 360.92 | (562.73) | 131.28 | (243.78) | 3.48 | 0.128 |
| R-COApm1-3 | 1131.67 | (749.32) | 1033.12 | (704.33) | 0.23 | 0.177 |
| R-PVHpml | 1014.42 | (568.81) | 811.76 | (426.40) | 2.00 | 0.138 |
| R-CUL4gr | 1769.83 | (1036.61) | 1465.52 | (836.56) | 1.28 | 0.138 |
| R-oct | 49.25 | (118.37) | 104.32 | (235.31) | 1.06 | 0.138 |
| R-VM | 383.00 | (264.81) | 252.24 | (229.48) | 3.42 | 0.128 |
| R-FLmo | 3.75 | (18.37) | 2.88 | (10.12) | 0.04 | 0.193 |
| R-VISal6a | 15.42 | (44.47) | 50.80 | (101.78) | 2.45 | 0.137 |
| R-SCdw | 15.58 | (45.85) | 7.36 | (36.80) | 0.48 | 0.157 |
| R-RSPagl6a | 0.00 | (0.00) | 0.00 | (0.00) | NaN | NaN |
| R-TR2 | 605.08 | (460.81) | 428.16 | (348.50) | 2.31 | 0.138 |
| R-FLgr | 28.92 | (79.58) | 55.60 | (202.47) | 0.36 | 0.162 |
| R-MPT | 108.08 | (123.91) | 132.24 | (106.39) | 0.54 | 0.157 |
| R-DR | 10.00 | (27.89) | 22.64 | (89.86) | 0.43 | 0.157 |
| R-AHNp | 291.00 | (351.83) | 135.60 | (148.04) | 4.12 | 0.119 |
| R-lotd | 0.00 | (0.00) | 0.00 | (0.00) | NaN | NaN |
| R-EPd | 36.33 | (86.70) | 38.96 | (75.55) | 0.01 | 0.199 |
| R-MPNm | 0.00 | (0.00) | 0.00 | (0.00) | NaN | NaN |
| R-INC | 205.75 | (336.21) | 369.76 | (398.52) | 2.41 | 0.137 |
| R-ECT6a | 172.42 | (234.63) | 463.76 | (413.31) | 9.10 | 0.057 |
| R-CENT3gr | 593.83 | (500.81) | 719.60 | (669.30) | 0.55 | 0.157 |
| R-cbt | 0.00 | (0.00) | 0.00 | (0.00) | NaN | NaN |
| R-ORB1 | 0.00 | (0.00) | 0.00 | (0.00) | NaN | NaN |
| R-AHNa | 1240.67 | (467.59) | 1658.56 | (566.62) | 7.89 | 0.057 |
| R-HPF | 47.83 | (96.44) | 20.64 | (59.84) | 1.42 | 0.138 |
| R-VAL | 63.17 | (81.14) | 18.32 | (50.88) | 5.42 | 0.086 |
| R-COAa | 463.33 | (535.14) | 433.68 | (314.08) | 0.06 | 0.193 |
| R-PARN | 123.67 | (451.04) | 27.52 | (90.89) | 1.09 | 0.138 |
| R-NOD | 41.92 | (110.43) | 104.96 | (150.48) | 2.78 | 0.133 |
| R-RSPd | 549.33 | (556.56) | 663.20 | (603.74) | 0.47 | 0.157 |
| R-arb | 175.25 | (151.08) | 149.44 | (165.00) | 0.33 | 0.166 |
| R-IV | 70.25 | (112.50) | 23.60 | (62.50) | 3.25 | 0.128 |
| R-AUDv | 83.25 | (171.57) | 75.04 | (118.20) | 0.04 | 0.193 |
| R-TTd1-4 | 0.00 | (0.00) | 0.00 | (0.00) | NaN | NaN |
| R-KF | 0.00 | (0.00) | 11.68 | (38.17) | 2.24 | 0.138 |
| R-DORpm | 367.33 | (448.60) | 146.64 | (128.80) | 5.58 | 0.086 |
| R-lab | 515.33 | (339.94) | 437.04 | (352.01) | 0.63 | 0.157 |
| R-ptf | 383.33 | (649.36) | 420.72 | (550.27) | 0.05 | 0.193 |
| R-ttp | 244.25 | (280.43) | 295.92 | (270.21) | 0.43 | 0.157 |
| R-grv of CBX | 18.42 | (41.03) | 28.00 | (60.16) | 0.42 | 0.157 |
| R-VISpl6a | 414.42 | (287.70) | 393.44 | (386.53) | 0.05 | 0.193 |
| R-SSp-un | 450.17 | (590.15) | 580.96 | (678.96) | 0.52 | 0.157 |
| R-PBme | 557.92 | (453.55) | 893.12 | (487.76) | 6.19 | 0.081 |
| R-MH | 21.42 | (31.39) | 11.28 | (16.21) | 2.04 | 0.138 |
| R-IXn | 28.58 | (62.91) | 61.76 | (93.81) | 2.10 | 0.138 |
| R-VISpm4 | 475.58 | (301.86) | 261.84 | (246.84) | 7.39 | 0.057 |
| R-cbp | 630.83 | (599.12) | 570.88 | (721.87) | 0.10 | 0.193 |
| R-GU1 | 1620.58 | (808.60) | 1824.16 | (1084.96) | 0.55 | 0.157 |
| R-MSC | 198.00 | (472.86) | 246.88 | (498.56) | 0.12 | 0.191 |
| R-ORBm2/3 | 1338.67 | (479.73) | 1356.56 | (527.28) | 0.02 | 0.199 |
| R-SSp-bfd1 | 0.00 | (0.00) | 0.00 | (0.00) | NaN | NaN |
| R-DMHv | 194.92 | (209.09) | 125.52 | (229.94) | 1.22 | 0.138 |
| R-DECgr | 369.92 | (239.61) | 421.36 | (391.49) | 0.30 | 0.166 |
| R-CU | 311.33 | (259.18) | 409.84 | (316.21) | 1.42 | 0.138 |
| R-CA1sp | 329.92 | (195.18) | 274.32 | (172.88) | 1.12 | 0.138 |
| R-MO6a | 86.00 | (147.69) | 96.24 | (183.10) | 0.05 | 0.193 |
| R-VISam5 | 286.83 | (248.39) | 276.96 | (317.26) | 0.01 | 0.199 |
| R-CBXmo | 71.00 | (56.29) | 101.92 | (159.36) | 0.81 | 0.16 |
| R-PB | 116.17 | (111.20) | 163.76 | (195.24) | 1.09 | 0.14 |

[1] Wiring cost and topological participation of the mouse brain connectome. Mikail Rubinov, Rolf J. F. Ypma, Charles Watson, Edward T. Bullmore.

Proceedings of the National Academy of Sciences Aug 2015, 112 (32) 10032-10037; DOI: 10.1073/pnas.1420315112.

**Table S3. Node-wise comparisons of the nodal clustering coefficient of empirical connectomes.** Nodal features were compared between UPS and control conditions using ANOVAs, and p-values were corrected using the False Discovery Rate method. For details about each region, see [1].

| **Node name** | **UPS**  ***M (SD)*** | | **Control**  ***M (SD)*** | | ***F* statistic** | **adjusted *p*** |
| --- | --- | --- | --- | --- | --- | --- |
| L-AMd | 2382.42 | (4296.07) | 3330.98 | (5033.88) | 0.50 | 0.617 |
| L-SSp-m2/3 | 9444.70 | (6324.52) | 8335.60 | (5789.72) | 0.41 | 0.617 |
| L-AUDd6a | 795.39 | (3429.22) | 777.93 | (1824.84) | 0.00 | 0.742 |
| L-SSp-n4 | 7739.68 | (3797.77) | 8255.46 | (2864.69) | 0.29 | 0.639 |
| L-ml | 0.00 | (0.00) | 0.00 | (0.00) | NaN | NaN |
| L-AOBmi | 5395.62 | (5508.85) | 6181.20 | (5965.43) | 0.23 | 0.639 |
| L-TEa1 | 2090.09 | (4669.08) | 897.49 | (2225.92) | 1.32 | 0.568 |
| L-COApm1-3 | 3049.49 | (1301.17) | 3911.19 | (1501.48) | 4.59 | 0.377 |
| L-PVHpml | 3917.57 | (983.95) | 4729.15 | (1779.24) | 3.86 | 0.377 |
| L-CUL4gr | 3992.17 | (898.29) | 4257.43 | (838.68) | 1.14 | 0.568 |
| L-oct | 16115.26 | (11737.38) | 15469.10 | (9563.92) | 0.04 | 0.712 |
| L-VM | 7859.16 | (3751.14) | 7198.37 | (4472.96) | 0.31 | 0.639 |
| L-FLmo | 0.00 | (0.00) | 0.00 | (0.00) | NaN | NaN |
| L-VISal6a | 695.73 | (2369.47) | 945.76 | (2625.87) | 0.12 | 0.669 |
| L-SCdw | 358.90 | (1758.26) | 345.08 | (1725.38) | 0.00 | 0.742 |
| L-RSPagl6a | 0.00 | (0.00) | 0.00 | (0.00) | NaN | NaN |
| L-TR2 | 2135.69 | (1585.71) | 1721.95 | (1611.03) | 0.82 | 0.591 |
| L-FLgr | 5200.55 | (5863.57) | 3994.28 | (6062.03) | 0.50 | 0.617 |
| L-MPT | 6962.55 | (5594.77) | 8622.30 | (5094.86) | 1.18 | 0.568 |
| L-DR | 4715.41 | (7786.19) | 3722.85 | (6812.35) | 0.23 | 0.639 |
| L-AHNp | 6254.95 | (5490.42) | 7000.17 | (6014.68) | 0.20 | 0.639 |
| L-lotd | 287.58 | (1408.86) | 0.00 | (0.00) | 1.04 | 0.568 |
| L-EPd | 4911.49 | (6923.32) | 3712.71 | (6123.32) | 0.41 | 0.617 |
| L-MPNm | 0.00 | (0.00) | 0.00 | (0.00) | NaN | NaN |
| L-INC | 9813.13 | (3749.42) | 10862.61 | (3247.57) | 1.10 | 0.568 |
| L-ECT6a | 1011.03 | (1681.33) | 2249.71 | (3477.73) | 2.49 | 0.553 |
| L-CENT3gr | 7071.87 | (1657.83) | 6579.87 | (1326.66) | 1.32 | 0.568 |
| L-cbt | 0.00 | (0.00) | 408.18 | (2040.88) | 0.96 | 0.568 |
| L-ORB1 | 0.00 | (0.00) | 0.00 | (0.00) | NaN | NaN |
| L-AHNa | 3081.34 | (684.46) | 2919.27 | (653.64) | 0.72 | 0.617 |
| L-HPF | 9757.92 | (5489.24) | 12844.01 | (6074.18) | 3.47 | 0.377 |
| L-VAL | 3508.77 | (5616.97) | 2473.97 | (5077.26) | 0.46 | 0.617 |
| L-COAa | 5637.89 | (1315.95) | 5693.51 | (2432.76) | 0.01 | 0.725 |
| L-PARN | 0.00 | (0.00) | 449.76 | (2248.78) | 0.96 | 0.568 |
| L-NOD | 24507.04 | (7227.14) | 19900.91 | (9280.17) | 3.74 | 0.377 |
| L-RSPd | 5751.67 | (2530.68) | 5686.47 | (1729.78) | 0.01 | 0.725 |
| L-arb | 3041.01 | (2896.07) | 2541.88 | (2703.14) | 0.39 | 0.617 |
| L-IV | 5817.78 | (5227.17) | 6012.04 | (5586.10) | 0.02 | 0.725 |
| L-AUDv | 4521.84 | (4810.43) | 663.42 | (2296.57) | 13.01 | 0.065 |
| L-TTd1-4 | 0.00 | (0.00) | 0.00 | (0.00) | NaN | NaN |
| L-KF | 3386.40 | (5160.11) | 4353.52 | (4933.81) | 0.45 | 0.617 |
| L-DORpm | 3084.24 | (2208.73) | 2646.62 | (2401.73) | 0.44 | 0.617 |
| L-lab | 2360.54 | (1567.74) | 2515.74 | (1409.07) | 0.13 | 0.669 |
| L-ptf | 2620.80 | (2933.91) | 2532.97 | (3426.90) | 0.01 | 0.725 |
| L-ttp | 1653.41 | (1588.63) | 1895.26 | (2160.72) | 0.20 | 0.639 |
| L-grv of CBX | 9970.86 | (3632.21) | 10361.24 | (3027.91) | 0.17 | 0.656 |
| L-VISpl6a | 6171.33 | (2845.79) | 6265.27 | (3060.32) | 0.01 | 0.725 |
| L-SSp-un | 7400.23 | (2703.64) | 8285.07 | (3083.96) | 1.14 | 0.568 |
| L-PBme | 4681.33 | (1589.51) | 4304.49 | (1145.34) | 0.91 | 0.568 |
| L-MH | 2958.32 | (4049.81) | 2480.41 | (3442.04) | 0.20 | 0.639 |
| L-IXn | 10622.21 | (5832.64) | 9735.53 | (5867.40) | 0.28 | 0.639 |
| L-VISpm4 | 5184.27 | (1648.97) | 5943.12 | (1290.53) | 3.23 | 0.377 |
| L-cbp | 7140.49 | (1578.66) | 7560.91 | (1532.23) | 0.89 | 0.568 |
| L-GU1 | 5408.71 | (923.56) | 5617.76 | (943.42) | 0.61 | 0.617 |
| L-MSC | 15473.52 | (2709.53) | 15387.66 | (2714.40) | 0.01 | 0.725 |
| L-ORBm2/3 | 3592.51 | (421.11) | 3681.75 | (564.04) | 0.39 | 0.617 |
| L-SSp-bfd1 | 0.00 | (0.00) | 0.00 | (0.00) | NaN | NaN |
| L-DMHv | 11282.16 | (2828.54) | 13002.10 | (3713.64) | 3.31 | 0.377 |
| L-DECgr | 14455.23 | (3788.96) | 15571.29 | (6119.64) | 0.58 | 0.617 |
| L-CU | 9314.03 | (1757.37) | 9645.55 | (1615.47) | 0.47 | 0.617 |
| L-CA1sp | 7119.17 | (1071.13) | 7682.55 | (1094.06) | 3.31 | 0.377 |
| L-MO6a | 13716.66 | (3345.12) | 13685.92 | (2205.55) | 0.00 | 0.742 |
| L-VISam5 | 9921.12 | (2335.64) | 11083.72 | (1985.09) | 3.54 | 0.377 |
| L-CBXmo | 12935.81 | (2103.80) | 14070.38 | (2013.43) | 3.72 | 0.377 |
| L-PB | 14119.35 | (3054.06) | 13152.61 | (2951.73) | 1.27 | 0.568 |
| R-AMd | 3701.08 | (4459.62) | 3091.47 | (4428.47) | 0.23 | 0.639 |
| R-SSp-m2/3 | 9532.76 | (5097.59) | 8733.66 | (5550.20) | 0.27 | 0.639 |
| R-AUDd6a | 1693.46 | (3032.30) | 763.35 | (1699.34) | 1.77 | 0.568 |
| R-SSp-n4 | 8978.69 | (3848.66) | 8983.61 | (4017.79) | 0.00 | 0.742 |
| R-ml | 0.00 | (0.00) | 0.00 | (0.00) | NaN | NaN |
| R-AOBmi | 4102.08 | (5682.03) | 3564.68 | (4807.66) | 0.13 | 0.669 |
| R-TEa1 | 1367.51 | (2849.80) | 662.09 | (1616.07) | 1.15 | 0.568 |
| R-COApm1-3 | 3075.13 | (1331.80) | 3305.81 | (1938.14) | 0.23 | 0.639 |
| R-PVHpml | 3954.76 | (830.06) | 4288.20 | (1243.42) | 1.21 | 0.568 |
| R-CUL4gr | 3866.75 | (1203.41) | 4252.88 | (1103.83) | 1.37 | 0.568 |
| R-oct | 11549.59 | (9904.63) | 14361.02 | (10830.22) | 0.90 | 0.568 |
| R-VM | 5782.65 | (2770.39) | 7673.34 | (3878.80) | 3.83 | 0.377 |
| R-FLmo | 1297.97 | (2997.05) | 638.43 | (2349.78) | 0.74 | 0.617 |
| R-VISal6a | 977.38 | (3504.95) | 2240.12 | (4155.40) | 1.32 | 0.568 |
| R-SCdw | 1362.48 | (3732.76) | 438.71 | (2193.56) | 1.13 | 0.568 |
| R-RSPagl6a | 0.00 | (0.00) | 0.00 | (0.00) | NaN | NaN |
| R-TR2 | 2036.23 | (1488.37) | 2181.72 | (2744.33) | 0.05 | 0.707 |
| R-FLgr | 7176.68 | (5770.09) | 4107.22 | (5647.50) | 3.54 | 0.377 |
| R-MPT | 8638.30 | (3829.41) | 8619.86 | (4754.27) | 0.00 | 0.742 |
| R-DR | 4119.65 | (7347.17) | 4429.66 | (6680.09) | 0.02 | 0.725 |
| R-AHNp | 3645.67 | (4090.60) | 4040.29 | (4930.12) | 0.09 | 0.685 |
| R-lotd | 0.00 | (0.00) | 0.00 | (0.00) | NaN | NaN |
| R-EPd | 5876.86 | (6839.26) | 3887.95 | (5424.38) | 1.28 | 0.568 |
| R-MPNm | 0.00 | (0.00) | 0.00 | (0.00) | NaN | NaN |
| R-INC | 9719.74 | (4251.36) | 9193.74 | (3853.82) | 0.21 | 0.639 |
| R-ECT6a | 2591.21 | (3412.42) | 1744.80 | (2659.91) | 0.94 | 0.568 |
| R-CENT3gr | 6726.99 | (1482.28) | 6089.15 | (1486.90) | 2.26 | 0.558 |
| R-cbt | 1285.38 | (4802.33) | 1141.40 | (3213.32) | 0.02 | 0.725 |
| R-ORB1 | 0.00 | (0.00) | 0.00 | (0.00) | NaN | NaN |
| R-AHNa | 3128.96 | (613.96) | 2769.68 | (420.22) | 5.76 | 0.377 |
| R-HPF | 10493.21 | (3193.69) | 12135.71 | (4389.73) | 2.23 | 0.558 |
| R-VAL | 6586.16 | (6267.64) | 2492.06 | (5094.32) | 6.32 | 0.377 |
| R-COAa | 5847.23 | (1901.23) | 5157.38 | (1511.05) | 1.99 | 0.568 |
| R-PARN | 488.28 | (2392.08) | 0.00 | (0.00) | 1.04 | 0.568 |
| R-NOD | 26597.79 | (10317.95) | 20289.22 | (9228.84) | 5.10 | 0.377 |
| R-RSPd | 7484.25 | (2907.32) | 6477.48 | (2576.86) | 1.65 | 0.568 |
| R-arb | 2462.94 | (3291.29) | 1900.57 | (2160.37) | 0.50 | 0.617 |
| R-IV | 4004.63 | (4769.21) | 3779.17 | (5318.35) | 0.02 | 0.725 |
| R-AUDv | 2432.39 | (3413.46) | 2107.12 | (3867.27) | 0.10 | 0.685 |
| R-TTd1-4 | 0.00 | (0.00) | 0.00 | (0.00) | NaN | NaN |
| R-KF | 3109.55 | (5029.18) | 4114.96 | (4831.01) | 0.51 | 0.617 |
| R-DORpm | 4139.96 | (1861.84) | 3710.72 | (2385.43) | 0.49 | 0.617 |
| R-lab | 2446.37 | (1330.61) | 2722.12 | (1005.16) | 0.67 | 0.617 |
| R-ptf | 2374.36 | (2248.62) | 2117.65 | (2378.66) | 0.15 | 0.664 |
| R-ttp | 3188.46 | (1845.93) | 2608.54 | (1647.92) | 1.35 | 0.568 |
| R-grv of CBX | 10788.19 | (3088.09) | 10726.53 | (3001.99) | 0.01 | 0.734 |
| R-VISpl6a | 5531.25 | (1941.72) | 5412.89 | (1375.63) | 0.06 | 0.705 |
| R-SSp-un | 7544.41 | (2498.13) | 8494.23 | (3502.47) | 1.19 | 0.568 |
| R-PBme | 4627.40 | (1380.30) | 4218.03 | (1173.64) | 1.25 | 0.568 |
| R-MH | 3377.57 | (3707.52) | 2077.77 | (2877.25) | 1.89 | 0.568 |
| R-IXn | 9966.68 | (6172.36) | 10989.43 | (5760.31) | 0.36 | 0.626 |
| R-VISpm4 | 5371.36 | (1200.81) | 6185.28 | (1314.74) | 5.11 | 0.377 |
| R-cbp | 7280.20 | (1414.57) | 7589.96 | (1430.48) | 0.58 | 0.617 |
| R-GU1 | 5313.26 | (1109.48) | 5174.20 | (975.92) | 0.22 | 0.639 |
| R-MSC | 16309.24 | (3153.72) | 14916.24 | (3180.58) | 2.37 | 0.558 |
| R-ORBm2/3 | 3869.24 | (508.89) | 3833.25 | (522.28) | 0.06 | 0.705 |
| R-SSp-bfd1 | 0.00 | (0.00) | 0.00 | (0.00) | NaN | NaN |
| R-DMHv | 11172.91 | (2558.82) | 12164.43 | (2848.68) | 1.64 | 0.568 |
| R-DECgr | 13561.29 | (4047.23) | 12935.16 | (2917.66) | 0.39 | 0.617 |
| R-CU | 9430.99 | (1626.14) | 8823.92 | (1730.58) | 1.60 | 0.568 |
| R-CA1sp | 7753.77 | (1296.95) | 8617.38 | (1446.23) | 4.83 | 0.377 |
| R-MO6a | 14129.66 | (2683.56) | 14627.95 | (2537.86) | 0.45 | 0.617 |
| R-VISam5 | 10391.02 | (1674.70) | 11876.82 | (2016.20) | 7.84 | 0.319 |
| R-CBXmo | 14346.41 | (3028.13) | 14156.70 | (1513.36) | 0.08 | 0.695 |
| R-PB | 14721.17 | (2895.77) | 13537.96 | (2847.78) | 2.08 | 0.568 |

[1] Wiring cost and topological participation of the mouse brain connectome. Mikail Rubinov, Rolf J. F. Ypma, Charles Watson, Edward T. Bullmore.

Proceedings of the National Academy of Sciences Aug 2015, 112 (32) 10032-10037; DOI: 10.1073/pnas.1420315112.

**Table S4. Node-wise comparisons of the nodal efficiency of empirical connectomes.** Nodal features were compared between UPS and control conditions using ANOVAs, and p-values were corrected using the False Discovery Rate method. For details about each region, see [1].

| **Node name** | **UPS**  ***M (SD)*** | | **Control**  ***M (SD)*** | | ***F* statistic** | **adjusted *p*** |
| --- | --- | --- | --- | --- | --- | --- |
| L-AMd | 2445.23 | (4365.49) | 3378.21 | (5053.51) | 0.48 | 0.633 |
| L-SSp-m2/3 | 10423.22 | (6759.70) | 9432.08 | (6139.11) | 0.29 | 0.648 |
| L-AUDd6a | 795.39 | (3429.22) | 848.38 | (2117.17) | 0.00 | 0.741 |
| L-SSp-n4 | 8984.69 | (3864.79) | 9651.40 | (3038.56) | 0.45 | 0.633 |
| L-ml | 0.00 | (0.00) | 0.00 | (0.00) | NaN | NaN |
| L-AOBmi | 5395.62 | (5508.85) | 6181.20 | (5965.43) | 0.23 | 0.665 |
| L-TEa1 | 2102.45 | (4668.55) | 1005.99 | (2374.73) | 1.09 | 0.584 |
| L-COApm1-3 | 4422.12 | (2421.30) | 5430.67 | (2290.48) | 2.25 | 0.536 |
| L-PVHpml | 6521.06 | (1873.79) | 7702.67 | (2105.03) | 4.29 | 0.371 |
| L-CUL4gr | 7082.74 | (1521.40) | 7872.15 | (1180.08) | 4.14 | 0.371 |
| L-oct | 16969.14 | (12082.79) | 16464.02 | (10107.72) | 0.03 | 0.739 |
| L-VM | 9232.97 | (4089.67) | 8540.52 | (5127.24) | 0.27 | 0.648 |
| L-FLmo | 0.00 | (0.00) | 0.00 | (0.00) | NaN | NaN |
| L-VISal6a | 695.73 | (2369.47) | 945.76 | (2625.87) | 0.12 | 0.703 |
| L-SCdw | 358.90 | (1758.26) | 345.08 | (1725.38) | 0.00 | 0.741 |
| L-RSPagl6a | 0.00 | (0.00) | 0.00 | (0.00) | NaN | NaN |
| L-TR2 | 2538.36 | (2145.77) | 1919.46 | (1865.35) | 1.16 | 0.584 |
| L-FLgr | 5200.55 | (5863.57) | 3994.28 | (6062.03) | 0.50 | 0.633 |
| L-MPT | 8253.29 | (5759.76) | 9519.99 | (4995.35) | 0.68 | 0.623 |
| L-DR | 4715.41 | (7786.19) | 3722.85 | (6812.35) | 0.23 | 0.665 |
| L-AHNp | 6568.45 | (5587.92) | 7270.67 | (6085.59) | 0.18 | 0.668 |
| L-lotd | 287.58 | (1408.86) | 0.00 | (0.00) | 1.04 | 0.584 |
| L-EPd | 5028.55 | (6951.74) | 3874.11 | (6259.95) | 0.37 | 0.648 |
| L-MPNm | 0.00 | (0.00) | 0.00 | (0.00) | NaN | NaN |
| L-INC | 11490.45 | (3792.18) | 12809.11 | (3098.04) | 1.78 | 0.584 |
| L-ECT6a | 1110.25 | (1891.41) | 2381.10 | (3682.31) | 2.28 | 0.536 |
| L-CENT3gr | 9468.38 | (1586.44) | 9039.37 | (1330.04) | 1.06 | 0.584 |
| L-cbt | 0.00 | (0.00) | 408.18 | (2040.88) | 0.96 | 0.584 |
| L-ORB1 | 0.00 | (0.00) | 0.00 | (0.00) | NaN | NaN |
| L-AHNa | 5828.25 | (1255.96) | 5599.83 | (1516.74) | 0.33 | 0.648 |
| L-HPF | 10992.58 | (5733.33) | 13791.79 | (6143.56) | 2.71 | 0.536 |
| L-VAL | 3508.77 | (5616.97) | 2473.97 | (5077.26) | 0.46 | 0.633 |
| L-COAa | 8956.64 | (1613.11) | 9017.48 | (2665.02) | 0.01 | 0.739 |
| L-PARN | 0.00 | (0.00) | 449.76 | (2248.78) | 0.96 | 0.584 |
| L-NOD | 25570.01 | (6391.51) | 21304.33 | (8222.13) | 4.09 | 0.371 |
| L-RSPd | 7164.93 | (2773.17) | 7236.87 | (2027.68) | 0.01 | 0.739 |
| L-arb | 3371.50 | (3189.39) | 2693.00 | (2990.61) | 0.59 | 0.623 |
| L-IV | 5943.12 | (5250.68) | 6012.04 | (5586.10) | 0.00 | 0.741 |
| L-AUDv | 4703.38 | (4931.80) | 663.42 | (2296.57) | 13.69 | 0.050 |
| L-TTd1-4 | 0.00 | (0.00) | 0.00 | (0.00) | NaN | NaN |
| L-KF | 3386.40 | (5160.11) | 4489.90 | (5020.68) | 0.58 | 0.623 |
| L-DORpm | 3649.21 | (2821.99) | 3063.53 | (2733.05) | 0.54 | 0.623 |
| L-lab | 2519.44 | (1633.01) | 2772.68 | (1586.41) | 0.30 | 0.648 |
| L-ptf | 2878.15 | (3286.93) | 2706.50 | (3551.73) | 0.03 | 0.739 |
| L-ttp | 1827.03 | (1780.17) | 2143.95 | (2301.43) | 0.29 | 0.648 |
| L-grv of CBX | 10830.46 | (3056.23) | 11054.09 | (2351.36) | 0.08 | 0.709 |
| L-VISpl6a | 8407.86 | (2485.72) | 8316.33 | (2416.07) | 0.02 | 0.739 |
| L-SSp-un | 8972.62 | (2881.36) | 9890.10 | (2936.23) | 1.22 | 0.584 |
| L-PBme | 7423.68 | (1967.52) | 7690.37 | (1527.42) | 0.28 | 0.648 |
| L-MH | 3172.29 | (4230.98) | 2683.35 | (3676.24) | 0.19 | 0.666 |
| L-IXn | 11001.63 | (5724.22) | 10503.34 | (5780.66) | 0.09 | 0.706 |
| L-VISpm4 | 6942.00 | (2103.71) | 7671.68 | (1225.41) | 2.22 | 0.536 |
| L-cbp | 10361.94 | (1543.01) | 10625.72 | (1333.76) | 0.41 | 0.640 |
| L-GU1 | 8582.23 | (1409.91) | 8995.68 | (1096.96) | 1.32 | 0.584 |
| L-MSC | 16429.50 | (2099.03) | 16035.09 | (2170.92) | 0.42 | 0.640 |
| L-ORBm2/3 | 7046.72 | (1112.58) | 7341.03 | (1150.02) | 0.83 | 0.623 |
| L-SSp-bfd1 | 0.00 | (0.00) | 0.00 | (0.00) | NaN | NaN |
| L-DMHv | 12557.11 | (2384.88) | 14064.38 | (2845.06) | 4.02 | 0.371 |
| L-DECgr | 18765.38 | (3884.30) | 19913.84 | (5130.46) | 0.78 | 0.623 |
| L-CU | 11489.81 | (1366.65) | 11559.26 | (1306.42) | 0.03 | 0.739 |
| L-CA1sp | 11708.69 | (1558.94) | 12502.35 | (1292.13) | 3.78 | 0.392 |
| L-MO6a | 15229.60 | (2827.07) | 15192.90 | (1869.86) | 0.00 | 0.741 |
| L-VISam5 | 12564.73 | (2204.76) | 13837.40 | (1825.43) | 4.86 | 0.371 |
| L-CBXmo | 15805.51 | (2183.10) | 16715.60 | (2039.73) | 2.28 | 0.536 |
| L-PB | 16769.68 | (2646.60) | 16143.65 | (2534.18) | 0.72 | 0.623 |
| R-AMd | 3874.33 | (4573.82) | 3297.88 | (4594.26) | 0.19 | 0.666 |
| R-SSp-m2/3 | 10747.31 | (5042.02) | 10143.88 | (5988.13) | 0.14 | 0.688 |
| R-AUDd6a | 1734.85 | (3098.42) | 763.35 | (1699.34) | 1.87 | 0.578 |
| R-SSp-n4 | 10378.89 | (3576.94) | 10200.25 | (3866.23) | 0.03 | 0.739 |
| R-ml | 0.00 | (0.00) | 0.00 | (0.00) | NaN | NaN |
| R-AOBmi | 4170.77 | (5686.62) | 3706.73 | (4926.90) | 0.09 | 0.706 |
| R-TEa1 | 1459.35 | (2921.10) | 758.63 | (1892.67) | 1.00 | 0.584 |
| R-COApm1-3 | 5029.44 | (2623.41) | 5050.92 | (2332.36) | 0.00 | 0.741 |
| R-PVHpml | 6921.67 | (1639.46) | 7286.15 | (1839.12) | 0.53 | 0.623 |
| R-CUL4gr | 7145.16 | (1767.93) | 7544.40 | (1781.48) | 0.62 | 0.623 |
| R-oct | 12558.01 | (10807.43) | 15394.16 | (11408.31) | 0.80 | 0.623 |
| R-VM | 7454.75 | (3358.19) | 9101.93 | (4586.19) | 2.04 | 0.539 |
| R-FLmo | 1297.97 | (2997.05) | 687.64 | (2459.86) | 0.61 | 0.623 |
| R-VISal6a | 977.38 | (3504.95) | 2303.03 | (4238.07) | 1.42 | 0.584 |
| R-SCdw | 1362.48 | (3732.76) | 438.71 | (2193.56) | 1.13 | 0.584 |
| R-RSPagl6a | 0.00 | (0.00) | 0.00 | (0.00) | NaN | NaN |
| R-TR2 | 2465.13 | (1955.41) | 2669.67 | (3534.37) | 0.06 | 0.728 |
| R-FLgr | 7176.68 | (5770.09) | 4107.22 | (5647.50) | 3.54 | 0.415 |
| R-MPT | 10148.12 | (3437.15) | 9099.12 | (4755.59) | 0.78 | 0.623 |
| R-DR | 4119.65 | (7347.17) | 4429.66 | (6680.09) | 0.02 | 0.739 |
| R-AHNp | 4063.73 | (4509.64) | 4526.66 | (5436.03) | 0.10 | 0.706 |
| R-lotd | 0.00 | (0.00) | 0.00 | (0.00) | NaN | NaN |
| R-EPd | 5876.86 | (6839.26) | 4041.59 | (5493.21) | 1.08 | 0.584 |
| R-MPNm | 0.00 | (0.00) | 0.00 | (0.00) | NaN | NaN |
| R-INC | 11418.88 | (4468.37) | 11311.88 | (3491.26) | 0.01 | 0.739 |
| R-ECT6a | 2932.77 | (3824.15) | 1914.33 | (2942.53) | 1.10 | 0.584 |
| R-CENT3gr | 9177.63 | (1620.75) | 8526.28 | (1452.25) | 2.20 | 0.536 |
| R-cbt | 1285.38 | (4802.33) | 1141.40 | (3213.32) | 0.02 | 0.739 |
| R-ORB1 | 0.00 | (0.00) | 0.00 | (0.00) | NaN | NaN |
| R-AHNa | 6135.97 | (1615.51) | 5153.24 | (1160.48) | 6.02 | 0.315 |
| R-HPF | 11960.33 | (2959.53) | 13203.74 | (4602.14) | 1.25 | 0.584 |
| R-VAL | 6586.16 | (6267.64) | 2492.06 | (5094.32) | 6.32 | 0.315 |
| R-COAa | 9275.14 | (2585.11) | 8453.43 | (1950.03) | 1.59 | 0.584 |
| R-PARN | 488.28 | (2392.08) | 0.00 | (0.00) | 1.04 | 0.584 |
| R-NOD | 27419.81 | (9912.16) | 22026.97 | (8141.15) | 4.35 | 0.371 |
| R-RSPd | 9233.48 | (2789.31) | 7914.70 | (2778.26) | 2.75 | 0.536 |
| R-arb | 2794.79 | (3717.00) | 2074.83 | (2435.60) | 0.65 | 0.623 |
| R-IV | 4114.98 | (4831.77) | 3845.67 | (5369.68) | 0.03 | 0.739 |
| R-AUDv | 2882.54 | (4062.64) | 2269.40 | (4073.02) | 0.28 | 0.648 |
| R-TTd1-4 | 0.00 | (0.00) | 0.00 | (0.00) | NaN | NaN |
| R-KF | 3109.55 | (5029.18) | 4292.93 | (5015.65) | 0.68 | 0.623 |
| R-DORpm | 4954.07 | (2323.89) | 4615.79 | (2843.84) | 0.21 | 0.665 |
| R-lab | 2823.91 | (1443.71) | 3104.68 | (1219.52) | 0.54 | 0.623 |
| R-ptf | 3009.30 | (3165.67) | 2514.20 | (2631.30) | 0.36 | 0.648 |
| R-ttp | 3816.40 | (2381.75) | 3081.26 | (2062.58) | 1.34 | 0.584 |
| R-grv of CBX | 11620.27 | (2342.74) | 11292.62 | (2662.56) | 0.21 | 0.665 |
| R-VISpl6a | 8058.07 | (1804.23) | 7569.84 | (1288.65) | 1.20 | 0.584 |
| R-SSp-un | 9429.72 | (2551.27) | 10211.77 | (3666.35) | 0.75 | 0.623 |
| R-PBme | 7546.00 | (2155.32) | 7519.61 | (1433.81) | 0.00 | 0.741 |
| R-MH | 3886.68 | (4174.04) | 2224.50 | (3097.45) | 2.52 | 0.536 |
| R-IXn | 10233.77 | (6140.74) | 11917.46 | (5612.96) | 1.00 | 0.584 |
| R-VISpm4 | 7360.03 | (1636.22) | 8052.93 | (1193.95) | 2.88 | 0.536 |
| R-cbp | 10555.04 | (1268.28) | 10493.05 | (1285.13) | 0.03 | 0.739 |
| R-GU1 | 8747.05 | (1268.02) | 8250.27 | (1082.29) | 2.18 | 0.536 |
| R-MSC | 17317.85 | (2645.17) | 15818.12 | (2370.43) | 4.38 | 0.371 |
| R-ORBm2/3 | 7722.07 | (886.35) | 7438.12 | (1092.08) | 0.99 | 0.584 |
| R-SSp-bfd1 | 0.00 | (0.00) | 0.00 | (0.00) | NaN | NaN |
| R-DMHv | 12639.11 | (2050.79) | 13525.08 | (2235.89) | 2.08 | 0.539 |
| R-DECgr | 18026.89 | (3561.67) | 17731.86 | (2670.58) | 0.11 | 0.706 |
| R-CU | 11590.03 | (1085.25) | 11147.95 | (1399.00) | 1.52 | 0.584 |
| R-CA1sp | 12278.74 | (2074.25) | 13760.21 | (1633.17) | 7.75 | 0.315 |
| R-MO6a | 15608.48 | (2227.52) | 16261.58 | (1849.04) | 1.25 | 0.584 |
| R-VISam5 | 13063.48 | (1809.15) | 14479.68 | (1997.99) | 6.75 | 0.315 |
| R-CBXmo | 16990.58 | (2866.13) | 16912.96 | (1533.72) | 0.01 | 0.739 |
| R-PB | 17475.07 | (2456.13) | 17094.31 | (2441.42) | 0.30 | 0.648 |

[1] Wiring cost and topological participation of the mouse brain connectome. Mikail Rubinov, Rolf J. F. Ypma, Charles Watson, Edward T. Bullmore.

Proceedings of the National Academy of Sciences Aug 2015, 112 (32) 10032-10037; DOI: 10.1073/pnas.1420315112.

**Table S5. Comparisons of the global topology of the empirical connectomes.** See Methods for details on the computation of the connectomic measures.

| **Measure** | **UPS**  **M (SD)** | **Control**  **M (SD)** | | **Test statistic*** | **p** |
| --- | --- | --- | --- | --- | --- |
| **Edge count** | 296.875 (10.250) | | 297.320 (11.943) | F_1,47_ = 0.02 | 0.89 |
| **Long-distance connections** | 20.583 (6.199) | | 19.360 (6.945) | D_1,47_ = 0.42 | 0.52 |
| **Maximum modularity** | 0.591 (0.025) | | 0.589 (0.022) | F_1,47_ = 0.13 | 0.72 |
| **System segregation** | 0.593 (0.177) | | 0.496 (0.403) | D_1,47_ = 0.26 | 0.32 |
| **Global efficiency** | 0.240 (0.015) | | 0.240 (0.013) | D_1,47_ = 0.19 | 0.71 |
| **Small-worldness** | 4.200 (0.395) | | 4.112 (0.445) | F_1,47_ = 0.54 | 0.47 |
| * A Shapiro test was applied to test the normality of the distributions. Normal distributions were compared using ANOVA, while others were compared using a KS test.  Note. “UPS” = unpredictable postnatal stress. | | | | | |

**Table S6. Comparisons of the distributions of local topology of the empirical connectomes.** Distributions of local characteristics, taken as the group average for each node, were compared between UPS and control conditions using Kolmogorov-Smirnov (KS) tests. See Methods for details on the computation of the connectomic measures.

| **Measure** | **UPS**  **M (SD)** | **Control**  **M (SD)** | | **D statistic** | **p** |
| --- | --- | --- | --- | --- | --- |
| **Node degree** | 1.292 (0.859) | | 1.200 (0.913) | 0.046 | 1.00 |
| **Betweenness centrality** | 2.476 (4.959) | | 1.204 (4.670) | 0.054 | 0.99 |
| **Clustering coefficient** | 0.236 (0.423) | | 0.320 (0.456) | 0.062 | 0.96 |
| **Edge length** | 29.743 (19.116) | | 28.159 (21.694) | 0.039 | 1.00 |
| **Mean matching index** | 0.022 (0.017) | | 0.019 (0.016) | 0.062 | 0.96 |
| **Nodal efficiency** | 0.243 (0.431) | | 0.327 (0.463) | 0.062 | 0.96 |
| Note. “UPS” = unpredictable postnatal stress. | | | | | |

**Table S7. Energy, optimal parameters, and topological dissimilarity for each generative rule.** Lowest-energy networks for each animal (N = 49) were obtained by comparing 160,000 combinations of parameters in the space defined by $-10\leq\eta\leq0$ and $0\leq\gamma\leq10.$

| Generative Model | Energy | | Eta $\boldsymbol{(\eta)}$ | | Gamma ($\boldsymbol{\gamma)}$ | |
| --- | --- | --- | --- | --- | --- | --- |
|  | **Mean** | **CV** | **Mean** | **CV** | **Mean** | **CV** |
| Spatial | 0.309 | 6.848 | -4.185 | -12.314 | 5.120 | 52.631 |
| Neighbour | 0.103 | 12.562 | -2.667 | -9.817 | 0.360 | 8.488 |
| Match | 0.110 | 11.276 | -2.624 | -9.842 | 0.402 | 11.236 |
| C-Avg | 0.170 | 11.972 | -7.301 | -13.144 | 2.361 | 34.19 |
| C-Min | 0.214 | 8.094 | -5.586 | -22.763 | 0.546 | 22.261 |
| C-Max | 0.178 | 12.184 | -8.36 | -15.669 | 4.946 | 52.494 |
| C-Diff | 0.207 | 10.056 | -6.166 | -8.732 | 0.880 | 24.858 |
| C-Prod | 0.215 | 8.176 | -5.686 | -14.933 | 0.569 | 15.848 |
| D-Avg | 0.098 | 9.357 | -4.787 | -13.923 | 2.596 | 12.808 |
| D-Min | 0.157 | 9.426 | -5.116 | -11.233 | 0.435 | 13.393 |
| D-Max | 0.092 | 8.806 | -4.968 | -15.855 | 2.732 | 15.693 |
| D-Diff | 0.132 | 10.305 | -5.514 | -25.623 | 2.507 | 21.68 |
| D-Prod | 0.155 | 8.889 | -5.000 | -11.119 | 0.379 | 14.452 |

*Note.* “$\Delta TF$” = topological fingerprint dissimilarity, *:* “Neighbour” = Number of Shared Neighbours, “Match” = Matching Index, “C-Avg” = Average Clustering, “C-Min” = Minimum Clustering, “C-Max” = Maximum Clustering, “C-Diff” = Difference in Clustering, “C-Prod” = Product of Clustering, “D-Avg” = Average Degree, “D-Min” = Minimum Degree, “D-Max” = Maximum Degree, “D-Diff” = Difference in Degree, “D-Prod” = Product of Degree.

**
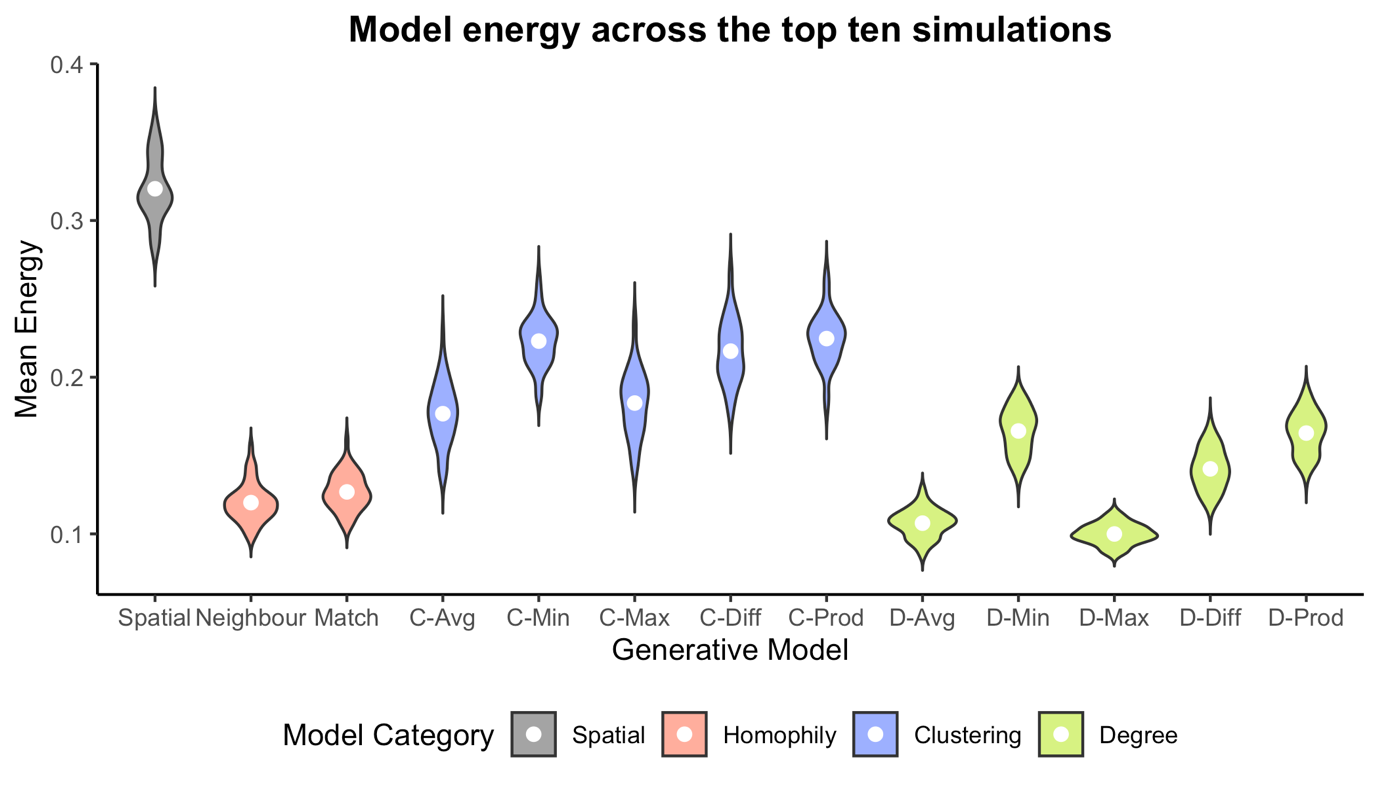
Figure S2. Relative performance of generative network models across the top ten simulations*.*** The energy of the best-performing synthetic networks for each animal (N = 49), averaged across the ten lowest-energy simulations, for thirteen generative rules: a purely spatial model, which considers only the distance between two regions; two homophily models, which also consider a measure of the similarity of the neighbourhoods of the respective regions; five clustering-based models, which compare the clustering coefficients of the regions; and five degree-based models, which compare their node degree. White points indicate the sample mean. *Note:* “Neighbour” = Number of Shared Neighbours, “Match” = Matching Index, “C-Avg” = Average Clustering Coefficient, “C-Min” = Minimum Clustering Coefficient, “C-Max” = Maximum Clustering Coefficient, “C-Diff” = Difference in Clustering Coefficient, “C-Prod” = Product of Clustering Coefficient, “D-Avg” = Average Degree, “D-Min” = Minimum Degree, “D-Max” = Maximum Degree, “D-Diff” = Difference in Degree, “D-Prod” = Product of Degree.


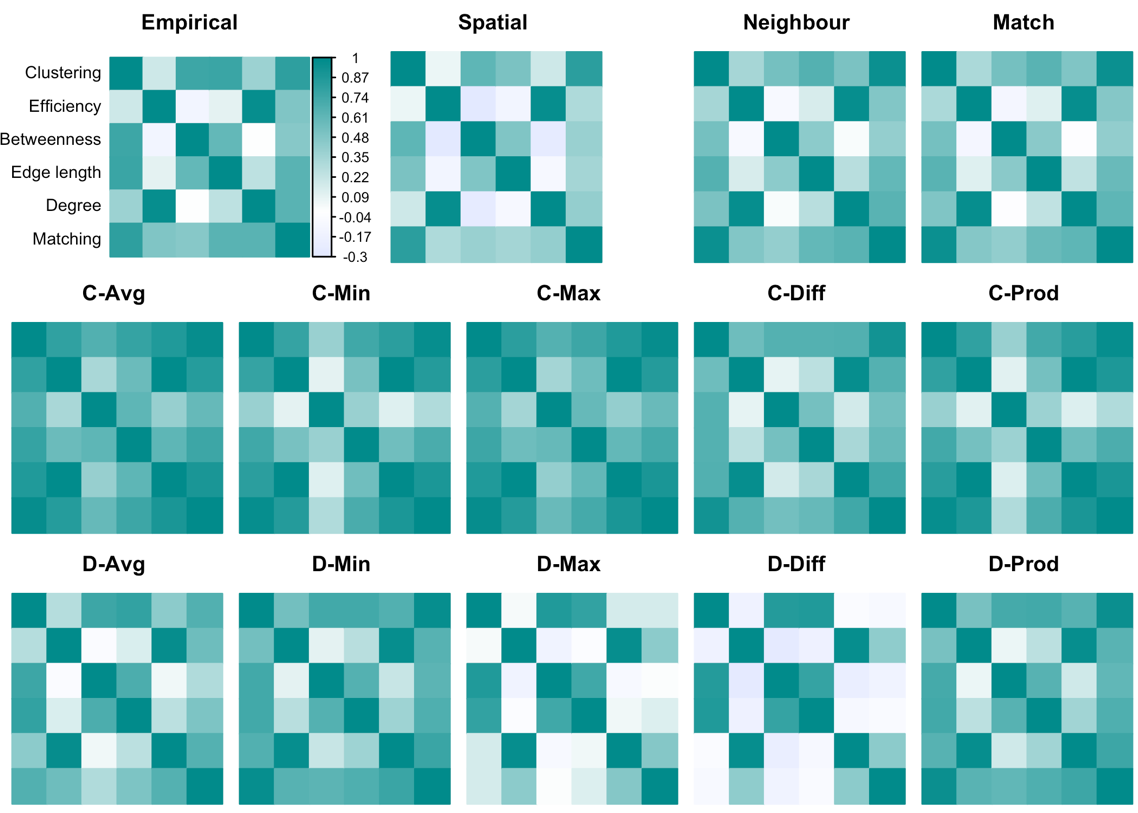

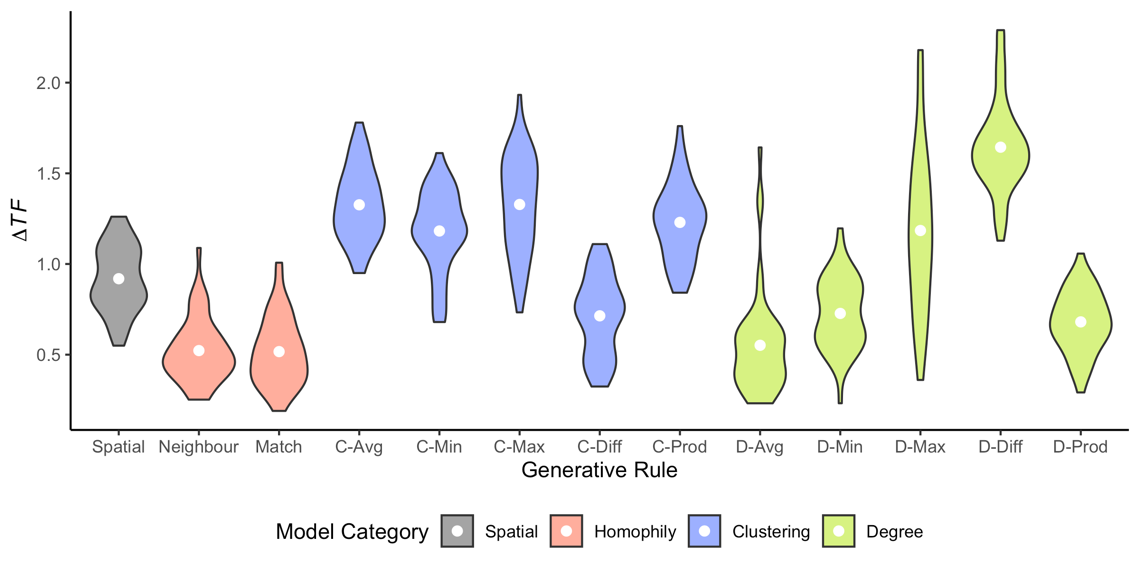


a

b

**Figure S3**. **Topological fingerprint matrices and topological dissimilarity for all generative models.** **(a)** The topological fingerprint is a correlation matrix of local network statistics, including node degree, clustering coefficient, betweenness centrality, total edge length, local efficiency and matching index. Across all matrices, the value of the correlation can be inferred from the colour bar, which spans -0.3 (pale lilac) through 0 (white) to 1 (teal). Correlations shown are the sample average (N = 49). For ease of visualisation, the measures are arranged according to the hierarchical clustering of measures in the empirical networks. **(c)** The neighbour model achieves lowest $\Delta TF$, a measure of discrepancy between synthetic and empirical patterns of connectivity. White points indicate the sample mean (N = 49). *Note:* “Neighbour” = Number of Shared Neighbours, “Match” = Matching Index, “C-Avg” = Average Clustering Coefficient, “C-Min” = Minimum Clustering Coefficient, “C-Max” = Maximum Clustering Coefficient, “C-Diff” = Difference in Clustering Coefficient, “C-Prod” = Product of Clustering Coefficient, “D-Avg” = Average Degree, “D-Min” = Minimum Degree, “D-Max” = Maximum Degree, “D-Diff” = Difference in Degree, “D-Prod” = Product of Degree.

**
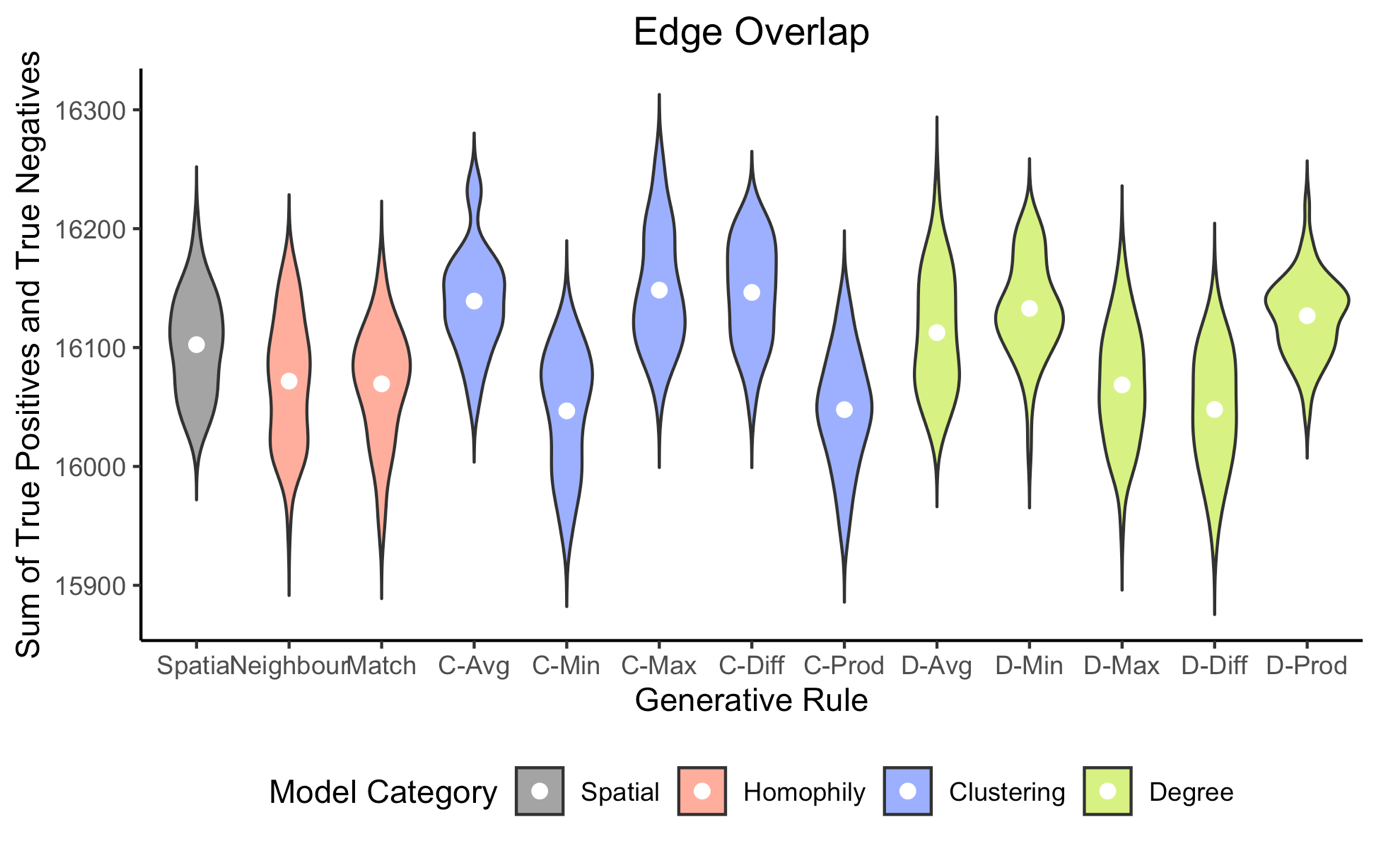
**

**Figure S4**. **Edge overlap between empirical connectomes and all generative models.** Edge overlap consists of the sum of true negatives and true positives, and was computed by comparing the empirical networks to the lowest-energy simulations for each subject across models. White points indicate the sample mean (N = 49). *Note:* “Neighbour” = Number of Shared Neighbours, “Match” = Matching Index, “C-Avg” = Average Clustering Coefficient, “C-Min” = Minimum Clustering Coefficient, “C-Max” = Maximum Clustering Coefficient, “C-Diff” = Difference in Clustering Coefficient, “C-Prod” = Product of Clustering Coefficient, “D-Avg” = Average Degree, “D-Min” = Minimum Degree, “D-Max” = Maximum Degree, “D-Diff” = Difference in Degree, “D-Prod” = Product of Degree.

**Table S8. Error across topological measures and nodes**. For each measure, the error quantifies the discrepancy observed between synthetic and empirical networks, while the absolute error is calculated as the sum of the Z-scores of all six errors. For details about each region, see [1].

| Node | Degree Error | Clustering Error | Betweenness Error | Edge Length Error | Efficiency Error | Matching Index Error | Absolute Error |
| --- | --- | --- | --- | --- | --- | --- | --- |
| L-AMd | -0.22 | 0.14 | -37.18 | -3.42 | 0.12 | 0.01 | 1.42 |
| L-SSp-m2/3 | -0.53 | 0.24 | 25.21 | -2.46 | 0.22 | 0.01 | 2.13 |
| L-AUDd6a | -8.98 | -0.44 | -290.73 | -158.46 | -0.59 | -0.05 | 12.88 |
| L-SSp-n4 | 1.02 | 0.48 | -95.17 | 15.35 | 0.54 | 0.03 | 5.88 |
| L-ml | -3.63 | -0.34 | -64.12 | -73.31 | -0.41 | -0.03 | 7.08 |
| L-AOBmi | -1.92 | 0.06 | 69.14 | -43.88 | 0.01 | -0.02 | 2.67 |
| L-TEa1 | -1.82 | -0.22 | 25.6 | -28.2 | -0.28 | -0.01 | 3.66 |
| L-COApm1-3 | 5.88 | 0.14 | 534.87 | 114.14 | 0.24 | 0.05 | 8.59 |
| L-PVHpml | 4.31 | -0.2 | 811.1 | 48.52 | -0.23 | 0.01 | 6.08 |
| L-CUL4gr | 5.55 | -0.18 | 794.87 | 89.17 | -0.08 | 0.03 | 7.63 |
| L-oct | -2.37 | 0.11 | -273.33 | -46.5 | 0.04 | 0 | 3.16 |
| L-VM | 1.82 | 0.17 | 102.94 | 40.52 | 0.23 | 0.04 | 4.52 |
| L-FLmo | -1.02 | -0.16 | -91.14 | -29.43 | -0.17 | 0 | 2.83 |
| L-VISal6a | -1.04 | -0.08 | -66.69 | -26.06 | -0.1 | 0 | 2.18 |
| L-SCdw | -1.02 | -0.11 | -58.99 | -15.86 | -0.13 | 0.01 | 1.8 |
| L-RSPagl6a | -1.9 | -0.21 | -71.33 | -31.42 | -0.23 | -0.01 | 3.95 |
| L-TR2 | 3.16 | 0.01 | 508.69 | 39.5 | 0.01 | 0 | 3.17 |
| L-FLgr | 0.37 | 0.22 | -52.4 | -7.48 | 0.21 | 0.03 | 2.96 |
| L-MPT | -1.86 | 0.07 | -80.9 | -41.92 | 0.07 | 0.01 | 2.19 |
| L-DR | 0.29 | 0.2 | -29.77 | 5.42 | 0.19 | 0.02 | 2.6 |
| L-AHNp | -6.35 | 0.08 | -171.14 | -120.83 | -0.01 | -0.04 | 7.16 |
| L-lotd | -2.45 | -0.2 | -69.24 | -50.25 | -0.25 | -0.02 | 4.8 |
| L-EPd | -0.47 | 0.11 | -141.54 | 2.34 | 0.1 | 0.02 | 2.09 |
| L-MPNm | -2.41 | -0.29 | -59.58 | -45.5 | -0.34 | -0.02 | 5.32 |
| L-INC | -1.39 | 0.15 | -278.04 | -40.17 | 0.16 | 0.02 | 3.76 |
| L-ECT6a | -3.94 | -0.26 | 73.67 | -79.93 | -0.34 | -0.03 | 6.54 |
| L-CENT3gr | 2.84 | 0.12 | -39.92 | 58.21 | 0.22 | 0.04 | 4.93 |
| L-cbt | -1.78 | -0.2 | -102.34 | -23.13 | -0.23 | 0 | 3.3 |
| L-ORB1 | -10.43 | -0.56 | -257.05 | -128.4 | -0.72 | -0.07 | 14.55 |
| L-AHNa | 6.22 | -0.1 | 443.37 | 20.88 | -0.04 | 0.02 | 4.28 |
| L-HPF | -1.51 | 0.18 | -161.84 | -19.69 | 0.2 | 0.01 | 2.79 |
| L-VAL | -0.96 | 0.1 | -78.24 | -30.29 | 0.06 | 0.01 | 2.12 |
| L-COAa | 6.1 | -0.18 | 432.69 | 74.68 | -0.08 | 0.03 | 6.29 |
| L-PARN | -6.63 | -0.43 | -265.15 | -88.95 | -0.54 | -0.04 | 10.08 |
| L-NOD | -2.76 | 0.28 | -272.64 | -55.55 | 0.21 | -0.01 | 5.25 |
| L-RSPd | -0.47 | -0.29 | 319.89 | -44.03 | -0.36 | -0.02 | 5.41 |
| L-arb | -1.27 | -0.15 | -102.79 | -17.8 | -0.19 | -0.01 | 2.88 |
| L-IV | -1.39 | 0.24 | -76.28 | -22.73 | 0.2 | 0 | 2.98 |
| L-AUDv | -1.96 | -0.09 | -113.77 | -26.3 | -0.13 | -0.01 | 2.81 |
| L-TTd1-4 | -3.82 | -0.49 | -138.03 | -11.91 | -0.53 | -0.03 | 7.21 |
| L-KF | -0.98 | 0.24 | -84.92 | -13.53 | 0.22 | 0 | 2.8 |
| L-DORpm | 0.53 | 0.03 | -38.73 | 4.92 | 0.01 | 0 | 0.76 |
| L-lab | 1.53 | -0.02 | -9.48 | 21.55 | -0.04 | 0.01 | 1.49 |
| L-ptf | -4.16 | -0.16 | -206.16 | -49.74 | -0.25 | -0.03 | 5.87 |
| L-ttp | 0.51 | -0.03 | 174.81 | -6.98 | -0.07 | 0.01 | 1.12 |
| L-grv of CBX | -0.98 | 0.44 | -233.56 | -10.88 | 0.42 | 0.01 | 4.78 |
| L-VISpl6a | 3.47 | 0.11 | -116.26 | 33.41 | 0.22 | 0.03 | 4.3 |
| L-SSp-un | 1.86 | 0.3 | 69.48 | 22.8 | 0.36 | 0.04 | 4.92 |
| L-PBme | 7.71 | 0.05 | 430.47 | 27.26 | 0.19 | 0.03 | 5.84 |
| L-MH | 0.9 | 0.15 | -53.74 | -1.25 | 0.16 | 0.01 | 1.85 |
| L-IXn | -1.39 | 0.38 | -240.49 | -30.09 | 0.35 | 0.02 | 5.07 |
| L-VISpm4 | 3.57 | 0.22 | -10.2 | 6.38 | 0.34 | 0.04 | 4.71 |
| L-cbp | 4.08 | -0.02 | -2.32 | -2.05 | 0.09 | 0.03 | 2.68 |
| L-GU1 | 6.22 | -0.17 | 1230.54 | 4.3 | -0.08 | 0.04 | 7.39 |
| L-MSC | -2.84 | 0.38 | -486.32 | -53.82 | 0.29 | 0.01 | 5.98 |
| L-ORBm2/3 | 10.69 | -0.11 | 1647.14 | 75.78 | 0.02 | 0.04 | 11.06 |
| L-SSp-bfd1 | -5.67 | -0.47 | -335.72 | -88.6 | -0.59 | -0.03 | 9.97 |
| L-DMHv | -3 | 0.37 | -273.4 | -43.09 | 0.34 | 0 | 5.55 |
| L-DECgr | 1.37 | 0.18 | -273.17 | 15.76 | 0.25 | 0.03 | 4.13 |
| L-CU | -1.18 | 0.27 | -256.02 | -30.5 | 0.3 | 0 | 4.04 |
| L-CA1sp | 6.49 | 0.01 | 8.04 | 105.77 | 0.19 | 0.03 | 5.82 |
| L-MO6a | -0.8 | 0.33 | -221.42 | -28.66 | 0.29 | 0.01 | 3.96 |
| L-VISam5 | 2.55 | 0.02 | -45.18 | 37.45 | 0.08 | 0.01 | 2.34 |
| L-CBXmo | 0.84 | 0 | -95.62 | -19.04 | 0.03 | 0 | 1.1 |
| L-PB | 2.18 | -0.09 | 21.42 | 0.51 | -0.03 | 0.01 | 1.45 |
| R-AMd | 0.04 | 0.19 | -151.96 | 7.23 | 0.18 | 0.01 | 2.53 |
| R-SSp-m2/3 | -0.9 | 0.1 | -70.07 | 18.86 | 0.11 | 0.01 | 1.84 |
| R-AUDd6a | -7.88 | -0.34 | -263.05 | -51.13 | -0.49 | -0.04 | 9.16 |
| R-SSp-n4 | 1.53 | 0.48 | -67.28 | 40.02 | 0.55 | 0.03 | 6.65 |
| R-ml | -2.02 | -0.21 | -39.71 | -24.91 | -0.25 | -0.01 | 3.85 |
| R-AOBmi | -0.39 | -0.02 | 185.84 | 7.38 | -0.05 | 0 | 1.29 |
| R-TEa1 | -0.71 | -0.19 | 134.37 | 15.07 | -0.22 | 0 | 2.69 |
| R-COApm1-3 | 6.49 | 0.12 | 709.64 | 116.99 | 0.25 | 0.05 | 9.21 |
| R-PVHpml | 4.41 | -0.19 | 869.81 | 58.39 | -0.2 | 0.01 | 6.39 |
| R-CUL4gr | 6.69 | -0.14 | 891.72 | 115.2 | -0.03 | 0.04 | 8.68 |
| R-oct | -2.55 | 0.01 | -206.9 | -38 | -0.05 | 0 | 2.49 |
| R-VM | 3.31 | 0.21 | 201.61 | 75.8 | 0.31 | 0.05 | 6.72 |
| R-FLmo | -0.8 | 0 | -112.73 | -21.4 | -0.01 | 0 | 1.29 |
| R-VISal6a | -1.45 | -0.09 | -180.1 | -24.03 | -0.12 | -0.01 | 2.79 |
| R-SCdw | -2.06 | -0.19 | -89.53 | -24.39 | -0.23 | 0 | 3.24 |
| R-RSPagl6a | -1.61 | -0.11 | -92.3 | -19.35 | -0.12 | -0.01 | 2.71 |
| R-TR2 | 3.53 | 0.03 | 481.93 | 28.71 | 0.05 | 0.01 | 3.24 |
| R-FLgr | 0.2 | 0.3 | -24.57 | 1.81 | 0.29 | 0.02 | 3.28 |
| R-MPT | -2.22 | 0 | -158.84 | -28.32 | -0.02 | 0 | 1.88 |
| R-DR | -0.2 | 0.14 | -21.66 | -5.26 | 0.12 | 0.02 | 1.89 |
| R-AHNp | -6.29 | -0.19 | -94.75 | -50.31 | -0.28 | -0.04 | 6.96 |
| R-lotd | -1.08 | -0.09 | -36.35 | -13.27 | -0.1 | -0.01 | 1.97 |
| R-EPd | -1.16 | 0.13 | -105.45 | -0.28 | 0.09 | 0.02 | 2.14 |
| R-MPNm | -1.94 | -0.25 | -127.77 | -27.65 | -0.29 | -0.02 | 4.52 |
| R-INC | 0.04 | 0.11 | -102.35 | -16.94 | 0.15 | 0.02 | 2.46 |
| R-ECT6a | -2.49 | -0.15 | 25.07 | -25.98 | -0.2 | -0.02 | 3.65 |
| R-CENT3gr | 3.63 | 0.15 | -157.78 | 100.04 | 0.26 | 0.05 | 6.88 |
| R-cbt | -1.67 | -0.24 | -91.47 | -10.9 | -0.26 | 0 | 3.33 |
| R-ORB1 | -9.71 | -0.52 | -325.37 | -41.31 | -0.66 | -0.07 | 12 |
| R-AHNa | 7.41 | -0.17 | 733.8 | 39.9 | -0.13 | 0.01 | 6.35 |
| R-HPF | -1.76 | 0.23 | -220.68 | -24.71 | 0.25 | 0.01 | 3.53 |
| R-VAL | -1.31 | 0.04 | -80.58 | -28.72 | 0.01 | 0.01 | 1.74 |
| R-COAa | 5.84 | -0.07 | 201.38 | 78.08 | 0.04 | 0.03 | 5.12 |
| R-PARN | -7.02 | -0.46 | -192.47 | -14.42 | -0.59 | -0.04 | 8.73 |
| R-NOD | -2.49 | 0.22 | -249.33 | -11.57 | 0.15 | -0.01 | 3.56 |
| R-RSPd | 0.08 | -0.34 | 331.05 | 6.02 | -0.38 | -0.01 | 4.73 |
| R-arb | -1.67 | -0.31 | -56.07 | -12.93 | -0.35 | -0.01 | 4.17 |
| R-IV | -2.41 | -0.06 | -42.26 | -25.36 | -0.11 | 0 | 2.31 |
| R-AUDv | -2.39 | -0.18 | -168.6 | -21.52 | -0.21 | -0.01 | 3.72 |
| R-TTd1-4 | -4.2 | -0.44 | -154.67 | -10.37 | -0.5 | -0.03 | 7.03 |
| R-KF | -0.98 | 0.12 | -141.8 | -1.91 | 0.1 | 0 | 1.67 |
| R-DORpm | 1.24 | 0.06 | 23.94 | 47.52 | 0.08 | 0.01 | 2.08 |
| R-lab | 2.71 | -0.01 | 148.17 | 42.77 | -0.02 | 0.02 | 2.73 |
| R-ptf | -3.63 | -0.17 | -42.75 | 0.12 | -0.23 | -0.02 | 3.93 |
| R-ttp | 1.18 | 0.11 | 40 | 32.74 | 0.13 | 0.02 | 2.45 |
| R-grv of CBX | -1.06 | 0.57 | -248.01 | 4.01 | 0.55 | 0.02 | 6.06 |
| R-VISpl6a | 4.47 | 0.16 | -15.31 | 52.98 | 0.31 | 0.03 | 5.46 |
| R-SSp-un | 2.24 | 0.36 | 48.94 | 26.03 | 0.44 | 0.04 | 5.79 |
| R-PBme | 8.33 | 0 | 474.66 | 56.07 | 0.15 | 0.04 | 6.49 |
| R-MH | 0.59 | 0.12 | -63.39 | 3.49 | 0.13 | 0.01 | 1.66 |
| R-IXn | 0.02 | 0.49 | -148.9 | -10.61 | 0.47 | 0.03 | 5.27 |
| R-VISpm4 | 3.96 | 0.28 | -49.44 | 41.2 | 0.4 | 0.04 | 6.34 |
| R-cbp | 3.43 | -0.01 | -61.8 | 13.9 | 0.08 | 0.03 | 2.99 |
| R-GU1 | 6.88 | -0.15 | 1466.97 | 3.95 | -0.07 | 0.04 | 8.24 |
| R-MSC | -2.41 | 0.34 | -328.9 | -25.66 | 0.25 | 0.01 | 4.6 |
| R-ORBm2/3 | 8.88 | -0.27 | 1455.35 | 13.67 | -0.18 | 0.03 | 9.4 |
| R-SSp-bfd1 | -6.88 | -0.44 | -343.83 | -54.41 | -0.59 | -0.03 | 9.49 |
| R-DMHv | -3.41 | 0.27 | -344.47 | 9.02 | 0.24 | 0 | 4.42 |
| R-DECgr | 1.61 | 0.07 | -88.32 | 37.7 | 0.15 | 0.03 | 3.32 |
| R-CU | -1.43 | 0.21 | -264.79 | 27.95 | 0.23 | 0 | 3.67 |
| R-CA1sp | 6.02 | -0.06 | 87.33 | 103.27 | 0.09 | 0.03 | 5.54 |
| R-MO6a | -2.41 | 0.3 | -321.76 | 10.68 | 0.25 | 0 | 4.3 |
| R-VISam5 | 0.71 | -0.02 | -72.09 | 0 | -0.01 | 0 | 0.75 |
| R-CBXmo | 0.24 | 0 | -54.21 | 0 | 0.02 | 0 | 0.59 |
| R-PB | 2.06 | -0.06 | -46.92 | 0 | -0.01 | 0.01 | 1.32 |

[1] Wiring cost and topological participation of the mouse brain connectome. Mikail Rubinov, Rolf J. F. Ypma, Charles Watson, Edward T. Bullmore.

Proceedings of the National Academy of Sciences Aug 2015, 112 (32) 10032-10037; DOI: 10.1073/pnas.1420315112.

**Table S9. Correlations between error in spatial embedding and seed network characteristics.** The error in spatial embedding quantifies the discrepancy between the synthetic and empirical connectomes on one of six nodal characteristics: node degree, clustering coefficient, betweenness centrality, edge length, nodal efficiency, and matching index. For each characteristic, Pearson correlation coefficients were calculated between its value in the seed network and the mean spatial error across the sample.

| **Nodal characteristic** | **Correlation coefficient** | ***p* value** |
| --- | --- | --- |
| **Degree** | 0.4356 | 2.221 x 10^-7*^ |
| **Clustering** | -0.0104 | 0.9067 |
| **Betweenness** | 0.1167 | 0.1862 |
| **Edge length** | 0.1912 | 0.0293 |
| **Efficiency** | 0.0026 | 0.9767 |
| **Matching** | 0.0822 | 0.3523 |
| ** Correlation is significant at Bonferroni corrected p < 0.00833* | | |


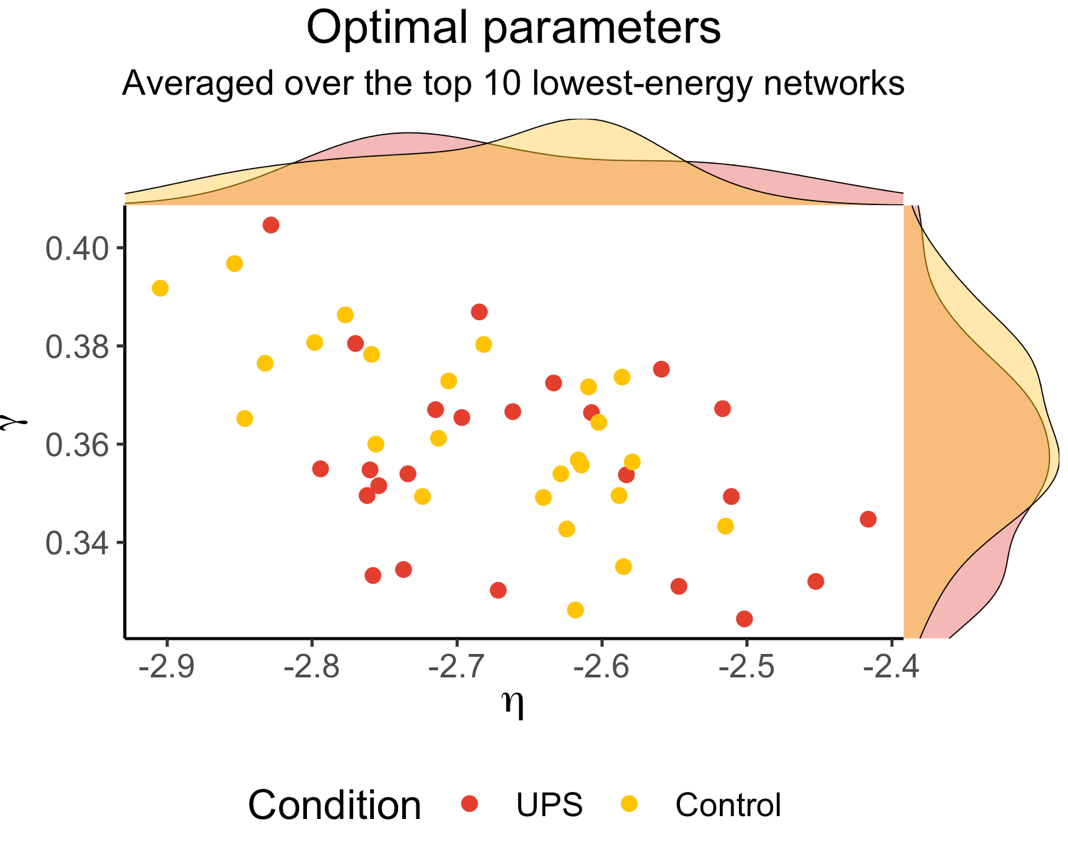


**Figure S5. Optimal generative modelling parameters averaged across the top ten networks.** Values of η and γ that produce the ten lowest-energy simulations were obtained by testing 40,000 parameter combinations in a narrow low-energy window of the initial grid search. Parameters producing the ten lowest-energy simulations were then averaged. Each data point in the scatterplot represents the parameters for a single animal. Density plots above and to the right compare UPS and control conditions.

**
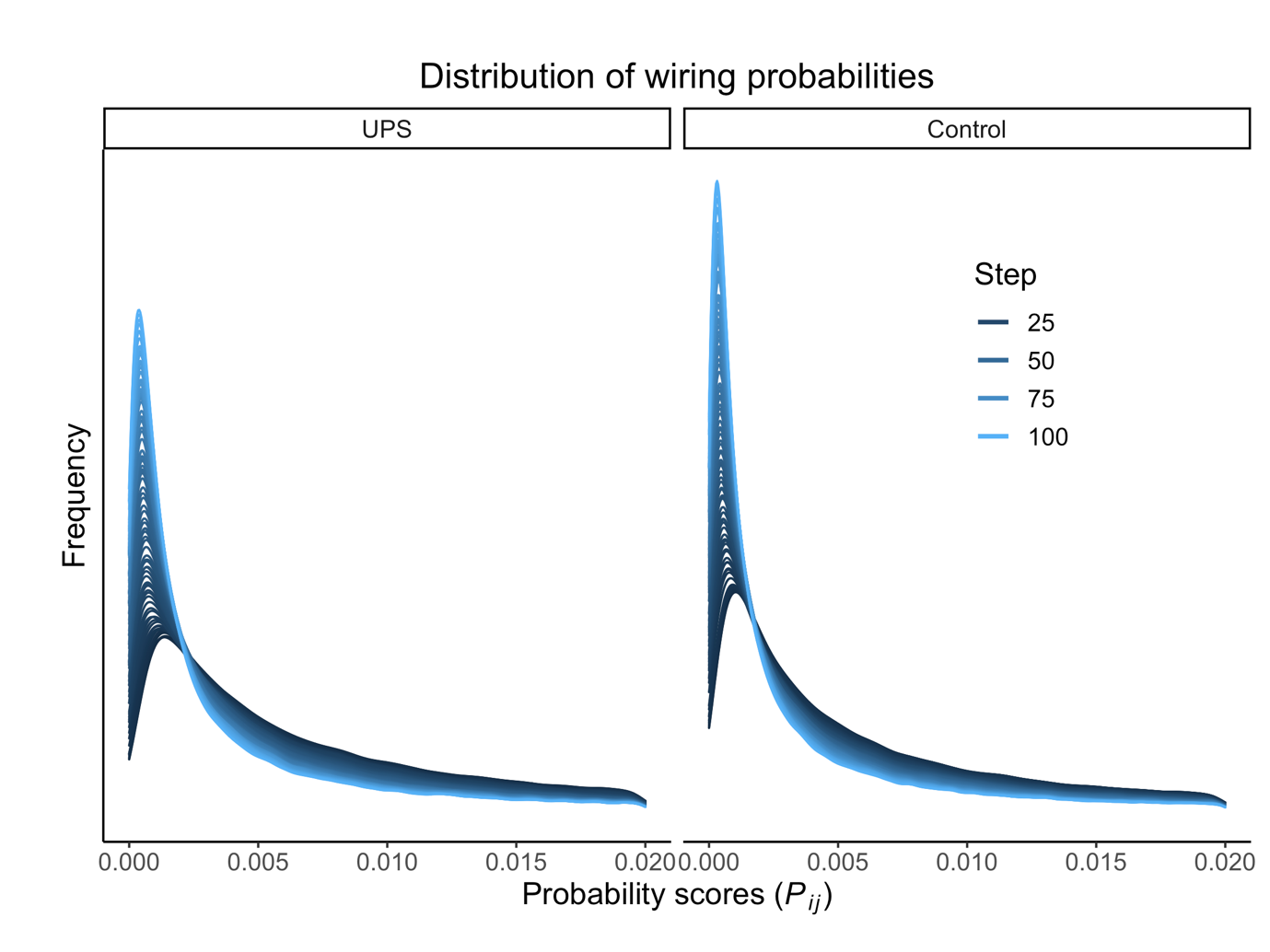
Figure S6. Distribution of wiring probabilities across the generative process.** Distributions of wiring probabilities ($P_{i,j}$) within the probability matrix, taken as the group averages. The colour of the curves corresponds to the step of the generative process, ranging from the start (0; dark blue) to the end (100; light blue). Across the development of the simulations, the UPS condition shows flatter distributions with greater dispersion, corresponding to more connections with higher wiring probabilities.

**
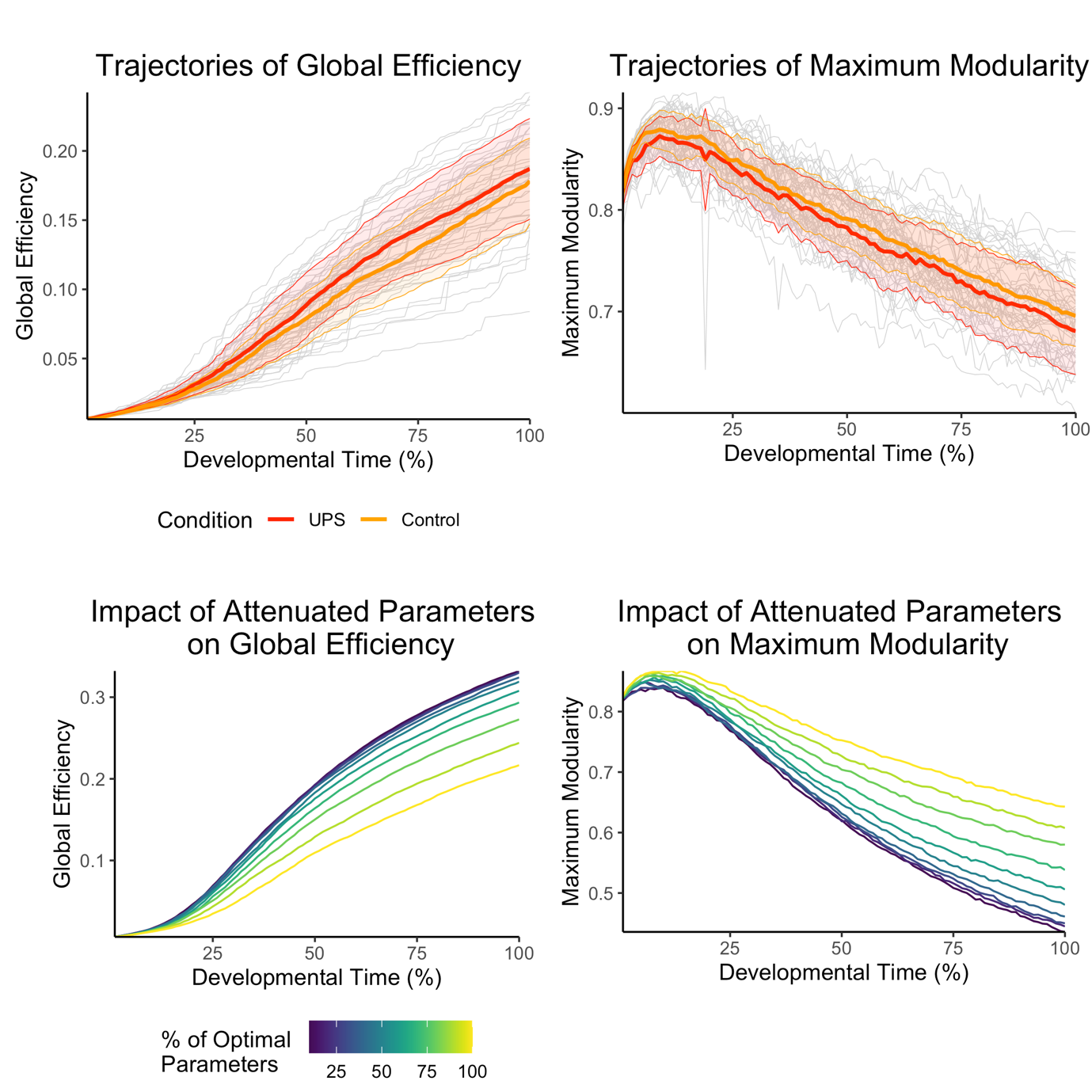
**

a b

c d

**Figure S7. Trajectories of emergence of integration and segregation across the generative process. (a-b)** Each grey line represents the trajectory of a single subject (N = 49) while the red and orange lines represent the group averages for the adversity and control conditions, respectively. The maximum modularity of the optimal networks **(a)** peaks early in development and subsequently declines. Optimal networks also exhibit stepwise gains in global efficiency **(b)** across network development. **(c-d)** To assess the effect of systematically manipulating wiring constraints on the emergence of global topology, additional simulations were run at 10% increments from the optimal values for each animal (yellow) to zero (purple) (see **Methods** for additional detail). The attenuation of wiring parameters results in faster emergence of global efficiency **(c)** and loss of maximum modularity **(d)**.
